# Next-generation systematics: An innovative approach to resolve the structure of complex prokaryotic taxa

**DOI:** 10.1038/srep38392

**Published:** 2016-12-07

**Authors:** Vartul Sangal, Michael Goodfellow, Amanda L. Jones, Edward C. Schwalbe, Jochen Blom, Paul A. Hoskisson, Iain C. Sutcliffe

**Affiliations:** 1 Faculty of Health and Life Sciences, Northumbria University, Newcastle upon Tyne NE1 8ST, UK; 2 School of Biology, University of Newcastle, Newcastle upon Tyne NE1 7RU, UK; 3 Heinrich-Buff-Ring 58, Justus-Liebig-Universität, 35392 Gießen, Germany; 4 Strathclyde Institute of Pharmacy and Biomedical Sciences, University of Strathclyde, 161 Cathedral Street, Glasgow G4 0RE, UK

## Abstract

Prokaryotic systematics provides the fundamental framework for microbiological research but remains a discipline that relies on a labour- and time-intensive polyphasic taxonomic approach, including DNA-DNA hybridization, variation in 16S rRNA gene sequence and phenotypic characteristics. These techniques suffer from poor resolution in distinguishing between closely related species and often result in misclassification and misidentification of strains. Moreover, guidelines are unclear for the delineation of bacterial genera. Here, we have applied an innovative phylogenetic and taxogenomic approach to a heterogeneous actinobacterial taxon, *Rhodococcus*, to identify boundaries for intrageneric and supraspecific classification. Seven species-groups were identified within the genus *Rhodococcus* that are as distantly related to one another as they are to representatives of other mycolic acid containing actinobacteria and can thus be equated with the rank of genus. It was also evident that strains assigned to rhodococcal species-groups are underspeciated with many misclassified using conventional taxonomic criteria. The phylogenetic and taxogenomic methods used in this study provide data of theoretical value for the circumscription of generic and species boundaries and are also of practical significance as they provide a robust basis for the classification and identification of rhodococci of agricultural, industrial and medical/veterinary significance.

It is common knowledge that prokaryotes are widely distributed in nature though the lack of understanding about their abundance and the scale of their diversity feature among the major challenges facing microbiologists^1^,^2^,^3^. Prokaryotic systematics is a fundamental scientific discipline which, *inter alia*, provides the framework for determining the extent of diversity and underpins research into the ecological, industrial and medical importance of prokaryotes. Fundamental to the current practice of polyphasic taxonomy is the definition of taxa at different ranks in the taxonomic hierarchy. The term species, for instance, is generally defined as a group of closely related strains evolved from a common ancestor and which have a degree of phenotypic consistency, ≥70% pairwise DNA-DNA hybridization (DDH) values, ca. >98.7% identity between their 16S rRNA gene sequences and a high mutual phenetic similarity^4^,^5^,^6^. However, the value of phenotyping and DDH is limited, not least by a lack of reproducibility and compatibility of results between different laboratories^7^,^8^ while 16S rRNA gene sequences tend to provide insufficient resolution to distinguish between closely related species^9^,^10^. Moreover, it is not possible to apply the ‘polyphasic’ approach to unculturable bacteria^3^,^11^, the so called ‘microbial dark matter’.

The limitations of current approaches to prokaryotic systematics have been addressed by several workers who have pressed the need to embrace the genome^10^,^12^,^13^,^14^,^15^,^16^,^17^. Indeed, the advent of inexpensive whole genome sequencing technologies and associated bioinformatic tools are promoting a step change in taxonomic practice, notably the availability of new metrics for delineating species^10^,^12^,^18^. In contrast, prokaryotic genera remain loosely defined, typically based on monophyly of strains with an average sequence divergence <6% in16S rRNA gene phylogenies^3^. Only limited attempts have been made to define generic boundaries between prokaryotes^19^.

Here, we have applied a range of genomic approaches to clarify the taxonomy of the genus *Rhodococcus*; the long and chequered taxonomic history of this genus has been addressed in several authoritative reviews^20^,^21^,^22^. The genus is classified in the family *Nocardiaceae*^23^ of the order *Corynebacteriales*^24^. The former encompasses other mycolic acid containing taxa such as the genera *Gordonia, Nocardia, Smaragdicoccus* and *Williamsia* and the latter more distantly related genera including *Corynebacterium* and *Segniliparus*. The genus *Rhodococcus* currently contains nearly 50 species with validly published names which fall into several 16S rRNA gene lineages, notably ones corresponding to the *Rhodococcus corynebacterioides, Rhodococcus equi, Rhodococcus erythropolis* and *Rhodococcus rhodochrous* clades^25^,^26^. 16S rRNA phylogeny indicates the presence of up to nine distinct groups within this genus and highlights widespread taxonomic ambiguities within this taxon^27^. Furthermore, a number of gene clusters have been found to vary between major rhodococcal clades, emphasizing extensive variation at the genomic level^27^. Similarly, phylogenetic groups of rhodococcal species have been detected based on other genes, such as *alkB*^28^ and from the analysis of a limited number of rhodococcal genomes^29^. Thus, there is a clear need to further unravel taxonomic relationships within the genus *Rhodococcus*, particularly given the importance of *R. equi*, a facultative intracellular pathogen of animals, especially foals^30^, *Rhodococcus fascians*, a phytopathogen of numerous dicotyledonous plants^31^ and *R. erythropolis* which is capable of numerous industrially significant bioconversions and biodegradations^32^. To embed rhodococcal taxonomy within a genomic framework, we present here an analysis of 100 rhodococcal strains and 15 representatives from related genera. These analyses revealed the existence of seven species-groups within the genus *Rhodococcus* that are as distantly related to one another as they are to other *Corynebacteriales* genera, thereby confirming the need for a significant revision of rhodococcal systematics. These analyses also highlight widespread misclassification and misidentification of rhodococci within the genus. However, most importantly, the results of this study show that the taxogenomic approach has the potential to resolve complex taxonomic questions both at the intrageneric and supra-species (intra-family) level.

## Results

### *Rhodococcus*, a highly polyphyletic taxon

To investigate the genomic heterogeneity within *Rhodococcus*, we sequenced the genomes of 15 strains representing different taxa previously classified within the genus, including the type strains of “*Rhodococcus hoagii*” (priority type strain for *R. equi*^26^), *Rhodococcus corynebacterioides, Rhodococcus gordoniae, Rhodococcus kunmingensis, Rhodococcus kroppenstedtii, Rhodococcus opacus, Rhodococcus pyridinivorans, Rhodococcus phenolicus, Rhodococcus qingshengii, Rhodococcus ruber* and *Rhodococcus rhodochrous* (the type species of the genus), representatives from two previously identified *R. equi* subgroups^25^ and the unclassified strain *Rhodococcus* sp. AJR001. The genome sequences of 85 strains belonging to the genus *Rhodococcus* were retrieved from GenBank (July 2015), including two strains previously sequenced by us^33^,^34^ (Supplementary Table 1). We also included 15 publicly available genomes of representatives of related genera classified within the order *Corynebacteriales* both for comparative analyses and as outgroups (Supplementary Table 1). The resultant 115 genomes were re-annotated by the RAST pipeline^35^ to have an equivalence of annotation and were compared using EDGAR^36^ to calculate the core genome. Information on the size of assemblies, GC content and number of coding sequences, RNA genes and GenBank accession numbers is provided in Supplementary Table 1.

**Figure 1:**
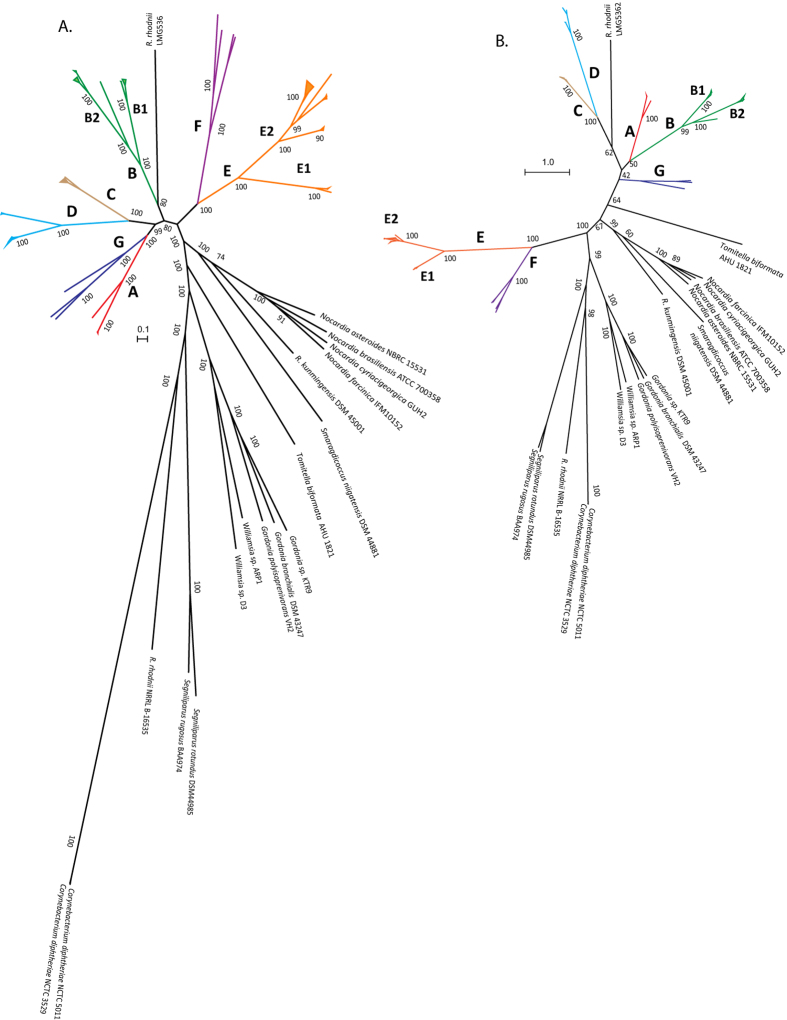
Un-rooted radial maximum-likelihood phylogenetic trees derived from (**A**) concatenated codon alignment of the core genome (scale bar represents nucleotide substitutions per codon site) and (**B**) a subset of amino acids from 400 broadly conserved prokaryotic proteins. The scale bar shows normalized fraction of total branch lengths as described by Segata *et al*.^38^.

A maximum-likelihood (ML) tree was constructed from a concatenated sequence alignment of codons from the core genes (255 genes) after stripping the start codons, stop codons as well as any codon with missing data using the best-fit codon substitution model (SCHN05 + F + I + G4). The rhodococci were clearly separated into seven distinct clusters and three singletons in the phylogenetic tree (Fig. 1A; Supplementary Fig. 1). *R. equi* formed a distinct group (group A) together with *R. defluvii*, a result consistent with those of previous analyses^26^,^33^. Despite its frequent association with *R. equi* in 16S rRNA gene trees^25^,^26^,^37^, the type strain of *R. kunmingensis* was recovered as a singleton that was loosely associated with group A in the phylogenetic tree.

The species assigned to the *R. rhodochrous* group (B, *Rhodococcus sensu stricto*) were subdivided into two major subgroups with the exception of the type strain of *R. phenolicus* which formed a phyletic line separate from each of the subgroups. In addition, *Rhodococcus triatomae* formed a distinct group (group G) together with two unclassified rhodococci (Fig. 1A; Supplementary Fig. 1). The two *Rhodococcus rhodnii* strains, symbionts in the gut of *Rhodnius prolixus*, a vector of Chagas disease, were also recovered as singletons; *R. rhodnii* NRRL B-16535^T^ was more closely related to *Corynebacterium diphtheriae* and *Segniliparus* strains than to other rhodococci. *Rhodococcus* species that formed sub-groups within the *R. erythropolis* clade in the 16S rRNA phylogeny of Jones *et al*.^25^ were separated into three distinct groups, C, D and a relatively distant group E. All of the *R. erythropolis* strains formed a single taxon, group D (Fig. 1A). The type strains of *R. corynebacterioides* and *R. kroppenstedtii* formed group F together with two unclassified rhodococci.

ML phylogenies were also reconstructed from a computationally selected subset of amino-acid sequences from 400 broadly conserved prokaryotic proteins^38^ (Fig. 1B; Supplementary Fig. 2) and the protein sequence alignment of the core genomes (Supplementary Fig. 3). Significantly, these trees confirm that the genus *Rhodococcus*, as presently defined, is polyphyletic and includes at least seven distinct species-groups. These results also show that 16S rRNA gene sequences have insufficient resolution to deduce precise inter-species relatedness within the genus *Rhodococcus*. A phylogenetic tree from 16S rRNA sequences extracted from the genomes confirms this conclusion (Supplementary Fig. 4).

### Taxogenomic separation of rhodococci into seven robust species-groups and identification of intrageneric and supraspecific boundaries

**Figure 2:**
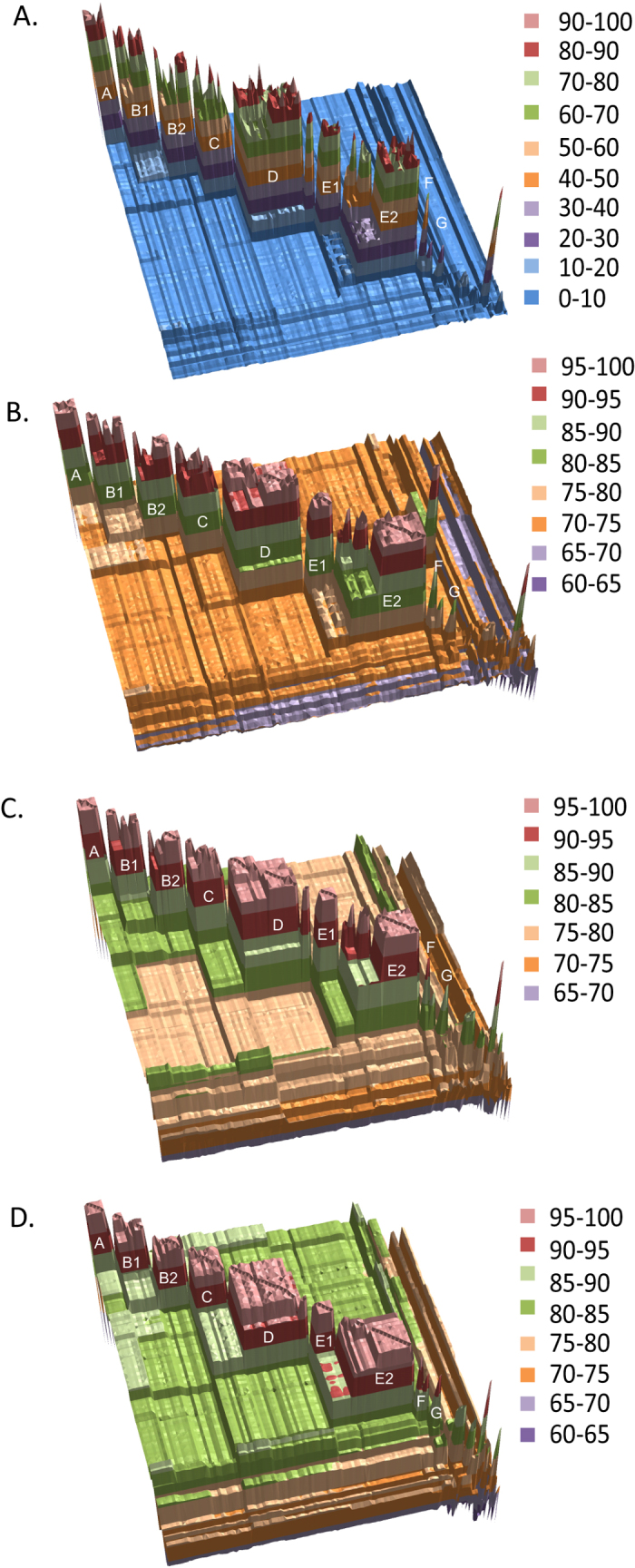
3D graphical representation of pairwise similarity matrices obtained by (**A**) fragmented BLAST searches (FBS values), (**B**) genomic average nucleotide identities (ANIb-G values), (**C**) average nucleotide identities among core genes (ANIb-C values) and (**D**) average amino-acid identities from the core genes (AAI values). *Rhodococcus* species-groups A-G are labelled whilst the reference genera are plotted at the lower right hand corner.

**Figure 3:**
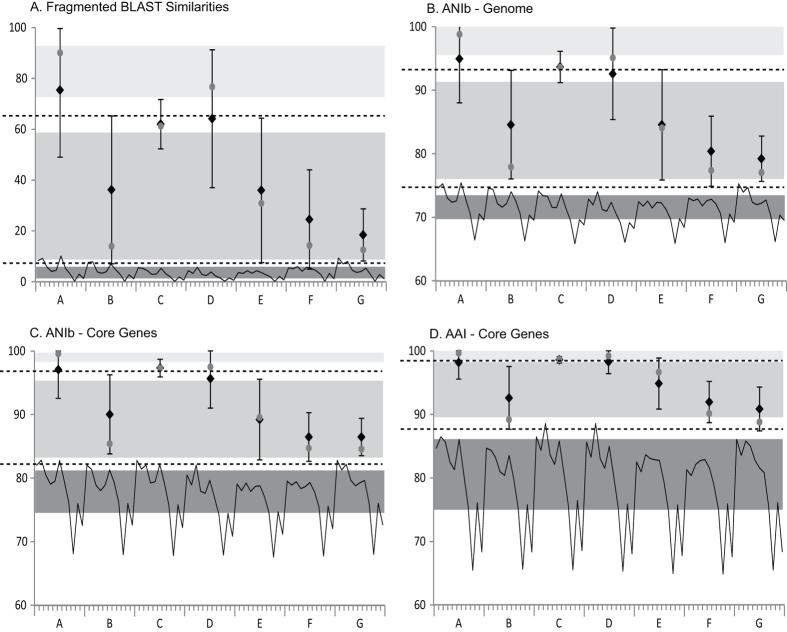
Average taxogenomic values (filled diamonds), (**A**) fragmented BLAST similarities (FBS), (**B**) genomic average nucleotide identities (ANIb-G), (**C**) average nucleotide identities among core genes (ANIb-C) and (**D**) average amino-acid identities from the core genes (AAI) with standard deviations. The median values are shown with filled circles. Average pairwise similarities with standard deviations within species, within groups of species (excluding similarity among members assigned to the same species), and between different groups are marked in light, intermediate and dark grey colour, respectively. The average diversity between different individual groups is plotted at the bottom for each group against species-groups (**A–G**), *Nocardia, Gordonia, Corynebacterium diphtheriae, Williamsia* and *Segniliparus*, respectively (excluding self-values).

The similarity matrix derived from the pairwise BLAST-based fragmented genome analysis supported the phylogenetic group structure (Figs 2A and 3A; Supplementary Table 2a). The mean similarity score (fragmented BLAST similarity, FBS) varied between 18.86 ± 10.26 and 75.82 ± 26.37 based on the diversity within each rhodococcal group. The pairwise similarity scores between the rhodococcal species-groups are between 2.49 ± 0.23 and 10.16 ± 0.50. However, two strains of group G showed slightly higher similarities (a score up to 11.31) with some strains in group A and *vice versa*.

These results are consistent with the BLAST-based pairwise average nucleotide identities from the whole genome sequences (ANIb-G). An ANIb-G value of ≥75% (79.20 ± 3.56–94.92 ± 6.92) was observed between strains within each of the species-groups, apart from group E where the values were marginally lower (down to 74.71%) between some strains (Figs 2B and 3B; Supplementary Table 2b). Similarly, the strains of species-group G share slightly higher ANIb-G values with the members of group A and *vice versa* (75.47 ± 0.33–75.33 ± 0.27). Multiple strains between rhodococcal species-groups A and B and groups A and C also showed >75% ANIb-G values. ANIb values calculated from the nucleotide sequences of the 255 core genes (ANIb-C) underlined the taxonomic integrity of these phylogenetic groups though similarity values were relatively higher than their corresponding ANIb-G values (Figs 2C and 3C; Supplementary Table 2c). The ANIb-C values within each rhodococcal species-group are >84% (86.39 ± 2.95–97.24 ± 1.39) though some strains from the two subgroups within group E showed slightly lower ANIb-C values (down to 81.1%).

An average amino acid identity (AAI) of 87.75–100% (mean, 90.85 ± 3.46–98.58 ± 0.50) was observed between two individuals of the same species-group from the core 255 protein sequences (Figs 2D and 3D; Supplementary Table 2d). The AAI values between different species-groups are <87% (80.42 ± 0.36–86.53 ± 0.17) except for groups C and D where they were slightly higher (88.60 ± 0.28).

The mean FBS score within a species was 83.45 ± 6.91 and between other members within a species-group 32.67 ± 24.29, resulting in a potential species threshold of 66.75 (Fig. 3A). A mean ANI-G value of 98.02 ± 0.84 was observed between strains within a species and corresponding 83.47 ± 7.30 values within each predicted genus suggesting a boundary of approximately 94% between species within the same genus (Fig. 3B). Although ANI-C and AAI values (99.11 ± 0.42 and 99.44 ± 0.76 within species and 89.28 ± 5.79 and 93.92 ± 4.22 within species-groups, respectively) clearly separated strains within and between species-groups (Fig. 3C,D), the species thresholds were much higher (96.88 and 98.41, respectively) with narrower buffer zones; hence, FBS and ANIb-G appear to be the most useful tools for delineating *Rhodococcus* species.

The mean FBS and ANIb-G values between different species-groups were found to be 3.64 ± 1.86 and 71.63 ± 1.86 with a suggested generic boundary of approximately 6.9 and 74.8, respectively (Fig. 3A,B). The ANI-C and AAI thresholds for delineating genera from the core genome were relatively higher that were around 82.3 and 87.8, respectively.

The taxogenomic similarities between members of the different *Rhodococcus* species-groups are comparable to corresponding similarities between these taxa and the representatives of the related genera (Figs 2A–D and 3A–D). Similar FBS scores were observed between the *Rhodococcus* species-groups (2.49 ± 0.23–10.16 ± 0.50) as between them and the genera *Gordonia, Nocardia* and *Williamsia* (1.51 ± 0.13–5.36 ± 0.84) while the *C. diphtheriae* and *Segniliparus* strains have relatively distant values (0.13 ± 0.05–1.23 ± 0.25). The ANIb-G, ANIb-C and AAI values between the rhodococcal species-groups are also comparable to those against the other genera though *C. diphtheriae* remains quite distant from all of these taxa (Figs 2A–D and 3A–D). These results clearly show that each of the rhodococcal species-groups can be considered to represent a distinct taxon (Fig. 2A–D and Fig. 3A–D; Supplementary Table 2a–d).

### Species-group A – *R. equi* cluster

**Figure 4:**
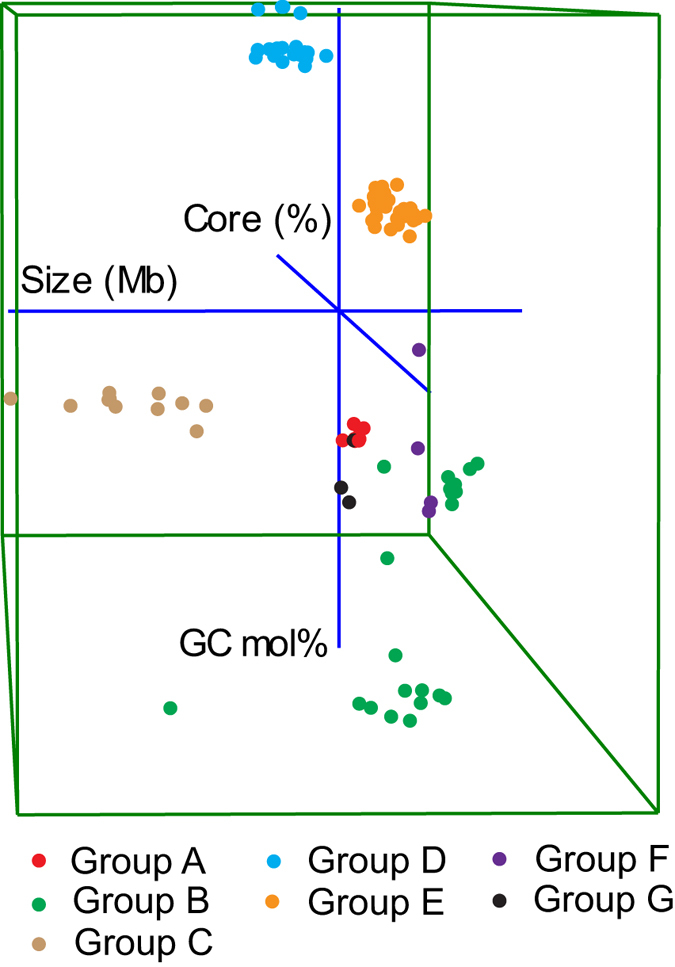
A 3D distribution of genome size, GC content and fraction of shared genes within each species-group (Supplementary Table 1). The three axes are shown in blue in the centre of the plot and are labelled. The individuals belonging to seven species-groups are shown in different colours.

The *R. equi* strains formed a distinct compact cluster together with the type strain of *R. defluvii*, a result consistent with our previous study^33^. These strains are primarily associated with foal disease and opportunistic human pathogenicity, with the exception of *R. defluvii.* The strains within this species-group shared 3,457 genes (63.6–73.5% of the total coding sequences). The genome size and GC content of these strains fell within the narrow range of 4.97–5.65 Mb and 68.5–68.8 mol%, respectively (Fig. 4). As expected, the *R. hoagii*/*R. equi* genomes are very closely related with a FBS similarity score of >82.6, ANIb-G values of >98.5% and ANIb-C and AAI values of >99.3% (Fig. 2A–D). Digital DNA-DNA hybridisation (dDDH, species cut-off ≥70%) values between these strains were >90.8% (Supplementary Table 3), these results are in line with the assignment of these strains to the same species^25^. ANIb-G values between the *R. equi* and *R. defluvii* strains are approximately 83%, and the corresponding ANIb-C and AAI values are ~89% and 93%, respectively. The dDDH value between the *R. hoagii/R. equi* strains and the *R. defluvii* strain is 27 ± 3%, a result consistent with their classification as separate species.

Jones *et al*.^25^ assigned *R. equi* strains to two subgroups based on the amplification of repetitive elements (*rep*-PCR), amplified 16S ribosomal DNA restriction analysis (ARDRA) and numerical taxonomic data^25^. However, phylogenetic and taxogenomic analyses (Figs 1 and 2A–D) of representatives of these subgroups (strains C7^T^ and N1288 from subgroup 1 and N1295 and N1301 from subgroup 2) did not show any evidence of subgroup structure (Figs 1A–B and 2A–D and Supplementary Figs 1–3), indicating that the division of strains into subgroups in the earlier analyses was probably more apparent than real.

### Species-group B – *Rhodococcus sensu stricto* cluster

This taxon can be considered to represent *Rhodococcus sensu stricto* as it includes the type strain of the type species of the genus, *R. rhodochrous* DSM 43241^T^. This species-group was found to encompass a diverse set of mainly environmental isolates which were divided into two subgroups, B1 and B2 (Fig. 1A,B and 2A–D). The mean FBS score within this species-group was 36.59 ± 29.08 and varied from 8.09–93.77 between individual pairs of strains. The average ANIb-G, ANIb-C and AAI values were 84.55 ± 8.54, 89.95 ± 6.21and 92.59 ± 4.93, respectively (Fig. 3A–D). The size of the genomes, average GC mol% and the shared fraction of genes within the group also show a clear subdivision of strains into the two subgroups (Fig. 4). However, two strains from subgroup B2, namely *R. rhodochrous* BKS6-46 and *Rhodococcus* sp. R4, had slightly larger genome sizes and varied in the fraction of the shared genes within the group (Supplementary Table 1).

Group B1 includes a *Rhodococcus aetherivorans* strain, five *R. ruber* strains, including the type strain, three unclassified rhodococci and a strain identified as *R. rhodochrous* (Fig. 1A,B, Supplementary Figs 1 and 2,Supplementary Table 1). The dDDH values (Supplementary Table 3) between representative strains of this taxon indicated the presence of three predicted species within this subgroup, a conclusion that is supported by pairwise ANIb-G and ANIb-C values (cut-off value of >94%; Supplementary Tables 1 and 2). The AAI from the core genes are >98% between individuals within these predicted species. One of the predicted species included four strains identified previously as *Rhodococcus* sp. BCP1, *Rhodococcus* sp. EsD8, *R. aetherivorans* IcdP1 and *R. rhodochrous* ATCC 21198 and a second one *R. ruber* strains DSM 43338^T^, IEGM 231, P25, Chol-4 and *Rhodococcus* sp. P14. The strain ‘*R. ruber*’ BKS 20–38 is clearly misidentified and may represent a third putative species given marginal taxogenomic similarities (ANIb genome, 94.76%; ANIb core genes, 97.21%; AAI core genes, 98.08%; dDDH, 60.3 ± 2.82) when compared with *R. ruber* DSM 43338^T^. The genome of strain BKS 20–38 is relatively large (6.13 Mb) and has a slightly lower GC content (69.7 mol%) compared with the other *R. ruber* strains (5.30–5.99 Mb; 70.2–70.7 mol%).

Group B2 includes the type strains of *R. rhodochrous, R. gordoniae* and *R. pyridinivorans* (Fig. 1A,B, Supplementary Figs 1 and 2, Supplementary Table 1), the taxogenomic analyses support the recognition of three species within this subgroup Supplementary Figs 1 and 2, (Supplementary Tables 1–3). The four unclassified strains in this subgroup can be assigned to known species. *Rhodococcus* sp. R1101 showed 85.7 ± 2.5 dDDH, >84 FBS score and >98% ANIb-G, ANIb-C and AAI similarities with *R. gordoniae* DSM 44689^T^ hence it can be assigned to this species. Similarly, taxogenomic values above the accepted species delineation thresholds were found between strains *Rhodococcus* sp. Chr-9, *Rhodococcus* sp. R4, *Rhodococcus* sp. P52 and *R. pyridinivorans* DSM 44555^T^. *R. phenolicus* DSM 44812^T^ does not belong to either of these subgroups (Fig. 1A,B), consistent with comparative taxogenomic values with individuals from each of the subgroups (ANIb genome, ANIb core genes and AAI core genes <90%, and dDDH between 21.9–23.0 ± 2.35; Supplementary Tables 1–3). ‘*R. rhodochrous*’ ATCC 21198 is taxogenomically distant from *R. rhodochrous* DSM 43241^T^ and is clearly misidentified.

### Species-group C – *Rhodococcus opacus* cluster

This group of environmental isolates encompasses eleven strains, five *Rhodococcus opacus*, two *Rhodococcus wratislaviensis*, one *Rhodococcus jostii*, one *Rhodococcus imtechensis* and two unclassified rhodococci (Supplementary Table 1). These strains were found to have genome sizes that varied between 7.8–10.4 Mb and GC contents between 66.8–67.9 mol% but nevertheless formed a distinct but diffused cluster based on both of these properties and on the fraction of shared genes (Fig. 4). dDDH values support the circumscription of four species within this group (Supplementary Table 3). The first of these taxa includes three strains, *R. jostii* RHA1, *Rhodococcus* sp. JVH1 and *Rhodococcus* sp. DK17, the second five strains, including *R. opacus* DSM 43205^T^, M213 and PD630, *R. wratislaviensis* IFP 2016 and *R. imtechensis* RKJ300^T^. The dDDH value between the type strains of *R. opacus* and *R. imtechensis* is 81.2 ± 2.7, the corresponding ANIb-G value >96% and the ANIb-C and AAI values ~98.9%, results which indicate that *R. imtechensis* RKJ300^T^ represents a later heterotypic synonym of *R. opacus* DSM 43205^T^. ‘*R. wratislaviensis*’ IFP 2016 is clearly misidentified as it is well separated from *R. wratislaviensis* NBRC 100605^T^ based on a dDDH value of 57.5 ± 2.8, a FBS score of <58 and an ANIb-G value of <93%; the ANIb-C and AAI values were ~97.0% and ~98.5%, respectively. ‘*R. opacus*’ strain R7 is also misclassified as the matrices show that it is a *bona fide R. wratislaviensis* strain. In turn, ‘*R. opacus*’ B4 probably represents a distinct species according to the taxogenomic analyses (Supplementary Tables 1–3). These results highlight the extent of misclassification and misidentification of rhodococcal strains thereby underlining the difficulty of classifying such strains reliably on the basis of traditional taxonomic criteria.

### Species-group D – *Rhodococcus erythropolis* cluster

This group of environmental isolates is compact and clearly defined based on genome size, GC content and the fraction of shared genes (Fig. 4). It encompasses 22 strains which fall into three species based on dDDH values (Supplementary Table 3), one of which includes four *R. erythropolis*, three *R. qingshengii* and two unclassified strains (Supplementary Tables 1 and 2). This taxon includes the type strain of *R. qingshengii* which shares a dDDH value of >80% with other strains (Supplementary Table 3). We obtained the partial sequences of 16S rRNA, *catA* and *gyrB* genes of this strain from GenBank (accession numbers DQ090961.1, KF500432.1 and KF374699.1, respectively) and confirmed the authenticity of the strain in a BLAST search that showed 100% coverage and identity with the sequenced *R. qingshengii* strain. The mean FBS score and ANIb-G values between the strains within this taxon were 83.04 and 98.01%, indicating that they may be reclassified as *R. qingshengii*.

Six strains classified as *R. erythropolis*, including the well-studied strain PR4, formed a second species within this species-group together with two strains of unclassified *Rhodococcus* spp., one of which was previously identified as *R. opacus* and the other as *R. rhodochrous* (Supplementary Tables 1–3). The 16S rRNA gene sequence of strain PR4 is identical to that of *R. erythropolis* DSM 43066^T^ (accession number: KJ476725.1). Hence the strains within this taxon belong to the species *R. erythropolis*. The third species within this group encompasses three strains, two of which have not previously been assigned species names while the remaining strain was described as *R. rhodochrous* (Supplementary Tables 1–3).

### Species-group E – *Rhodococcus fascians* cluster

The strains in this cluster can be divided into two subgroups based on phylogenetic and taxogenomic data (Figs 1, 2, 3 and Supplementary Tables 1–3), a result in line with an earlier report^29^. The strains within this taxon, which include plant pathogens and some environmental isolates, have a genome size ranging between 5.17–6.24 Mb and a fairly narrow GC content (64.1–64.7 mol%; Fig. 4). Seven strains, including two unclassified strains, formed a subgroup which corresponds to clade II as defined by Creason *et al*.^29^; this taxon encompasses two species (Supplementary Tables 1–3). The remaining sixteen *R. fascians* strains, including the type strain (LMG3623^T^), formed the second subgroup together with five unclassified strains (Supplementary Tables 1–3) that matches clade I in the above study^29^. These strains can be assigned to six predicted species based on dDDH, FBS score of >70 and ANIb-G values of >94%; five of these taxa correspond to taxa delineated by Creason *et al*.^29^.

### Minor *Rhodococcus* taxa

The remaining rhodococcal strains were assigned to two small groups, F and G and three singletons. Group F includes four strains, namely *R. corynebacterioides* DSM 20151^T^, *R. kroppenstedtii* DSM 44908^T^ and two unclassified strains, each representing a distinct species according to the taxogenomic data (Supplementary Tables 1–3). Group G encompasses three strains, *R. triatomae* BKS 15–14 and two unclassified rhodococci which belong to three predicted species (Supplementary Tables 1–3).

## Discussion

There is increasing evidence that current approaches to prokaryotic systematics will be enriched by the inclusion of whole genome sequencing data^10^,^12^,^13^,^14^,^15^,^16^,^17^. In particular, new metrics have been suggested for species delineation, as exemplified by an ANI cut-off of >94% and dDDH values of >70% to identify strains within a species^4^,^39^,^40^,^41^,^42^. A multi-gene phylogenetic approach applied to members of the class *Clostridia* indicated that they could be reclassified into multiple species that belonged to novel genera^43^. Here, we have built upon such studies by applying a comprehensive genomic approach to delineate species within the genus *Rhodococcus* that include strains of agricultural, industrial and medical/veterinary significance. The genetic heterogeneity within this genus has become increasingly clear, particularly in the light of a succession of 16S rRNA gene sequence analyses^21^,^22^,^25^,^27^,^44^. However, the number and composition of distinct lineages varied between these studies thereby indicating the need to re-examine relationships within this genus using genomic methods. Our genomic analyses of 100 *Rhodococcus* strains highlighted the presence of at least seven species-groups and three singletons (Fig. 1A,B; Supplementary Figs 1–3). It is particularly significant that these taxa are as distant from one another as they are from other genera classified in the family *Nocardiaceae* (Figs 2A–D and 3A–D) and should thus be recognised as putatively novel genera. These seven lineages were also identified in the 16S rRNA phylogeny from 641 most reliable sites (Supplementary Fig. 4); however, the resolution was very limited at the species level.

This integrated genomic approach identified clear intrageneric and supraspecific boundaries for a reliable delineation of species and genera (Fig. 3). FBS scores are average pairwise normalized BLAST similarity scores calculated using a non-overlapping 500 bp fragment size^45^. This approach is faster when a large number of genomes are compared. However, a more accurate matrix can be obtained using a computationally extensive approach with smaller fragment size and an overlapping sliding window. *Rhodococcus* species-groups, as well as different species within species-groups, are well separated using the FBS cut-off values of 6.9 and 66.75, respectively.

ANI was first calculated from the conserved genes for a robust resolution of prokaryotic species with minimum effect of horizontal gene transfer^42^. An ANI value of ~94% was suggested to correspond to an experimental DDH value of 70% for species separation. In this study, the ANIb-C threshold, calculated from 255 core genes, was relatively high (~96.88%) for species delineation. This value may be affected by the size of the core genome analysed, which is dependent on the number of genomes in the dataset as well as the criterion for defining orthologous genes. However, the approach of splicing the genome into 1020 bp fragments followed by BLAST-search against other genomes^46^ appears to be more pragmatic. The ANI is calculated from pairwise BLASTN matches with >30% sequence identity and ≥70% alignable length and an ANI value of 95% corresponds to the 70% DDH for species delineation^46^. The ANIb-G cut-off value to define rhodococcal species is ~94% (Fig. 3B), which is consistent with previous reports of defining an ANI cut-off of >94% to identify strains within a species^5^,^36^,^37^. The ANIb-G threshold for separating potential genera is ~74.8%.

It has been proposed that AAI derived from the conserved genes should be incorporated into prokaryotic taxonomy as AAI provides more robust resolution than ANI between divergent strains^47^,^48^. The AAI thresholds from the 255 core genes are 87.8% and 98.41% for separating potential genera and species, respectively. Again, these values may be affected by the number of genes in the core genome, as described for ANIb-C. The species designations with cut-off values from different matrices are also supported by dDDH values which are based on the genome to genome distance calculation that mimics the experiment based DDH values^39^,^40^. However, it will be important to use these taxogenomic indices and suggested thresholds in conjunction with robust genome based phylogenies.

Qin *et al*.^19^ suggested that ANI values are not suitable for separating genera^19^ and that a genus should be defined by a shared percentage of conserved proteins of at least 50%. Here, we have applied a more robust approach that uses fragmented BLAST similarity scores, ANI values and phylogenies assembled from universal proteins and the core genome, and found that ANIb-G values can reliably distinguish between rhodococci assigned to different species-groups. In contrast, the fraction of shared genes could be below 50% for diverse species-groups (Supplementary Table 1).

In this study, the genome sequences of 75 strains that represented 20 rhodococcal species, including 18 type strains, were analysed together with 25 strains that were unclassified at the species level (Supplementary Table 1). The taxogenomic analyses indicate that these strains should be classified into 31 species. Species-group E (*R. fascians*), the most underspeciated taxon, includes eight presumptive species thereby reinforcing previous work where strains classified as *R. fascians* were separated into different, albeit closely related species^29^. Similarly, some strains classified as *R. erythropolis, R. opacus, R. rhodochrous, R. ruber* and *R. wratislaviensis* were found to be sufficiently taxogenomically distinct to be separated into different species (Supplementary Tables 1–3). ‘*R. opacus*’ NRRL B-24011, ‘*R. rhodnii*’ LMG 5362, ‘*R. rhodochrous*’ ATCC 17895 and ‘*R. rhodochrous*’ NRRL B-1306 were also shown to be misclassified as they are more closely related to strains in taxonomically distinct species-groups than the corresponding type strains (Fig. 1A,B, Supplementary Table 1). The genomic analyses challenge the retention of *R. imtechensis* as a distinct species since the type strain of this taxon clearly belongs to the established species *R. opacus*. It is also significant that the taxogenomic approach allowed many of the unclassified strains to be assigned to validly published *Rhodococcus* species, as exemplified by the assignment of *Rhodococcus* strains BCP1 and EsD8 to *R. aetherivorans*, strain R1101 to *R. gordoniae*, strains Chr-9, R4 and P52 to *R. pyridinivorans*, strains JVH1 and DK17 to *R. jostii*, strain 311R to *R. erythropolis*, and strains PML 026 and JG-3 to *R. fascians*. Therefore, this study provides a proof of concept for the integration of genomics in prokaryotic systematics for a reliable, robust and stable classification of prokaryotic species.

Despite multiple calls to revisit complex rhodococcal taxonomy^20^,^21^,^22^,^25^,^27^,^29^, a recent study based on the analyses of fewer rhodococcal genome sequences presented an alternative phylogenomic view even though similar species-groups were recovered^49^. In contrast, the present study is based on more extensive and comprehensive phylogenomic and taxogenomic analyses of a larger genomic dataset, including more type strains. The results of this study clearly support the separation of rhodococci into multiple presumptive genera. The taxogenomic analyses (Figs 1 and 2) unequivocally support the proposal that *R. equi* be classified in the genus *Prescottella* as *Prescottella equi*^25^,^50^, and the subsequent conclusion that *R. defluvii* belongs to this taxon and should be classified as *Prescottella defluvii*^33^. Complex nomenclatural problems have delayed the formal validation of the names of these taxa^51^. Five genes that encode hypothetical proteins are specific to this presumptive novel genus according to BLAST searches in the NCBI nucleotide and protein sequence databases (Supplementary Table 4). In this context, it is interesting to note that *Myoviridae* phage E3 infects *R. equi* strains but not other rhodococci or mycolic acid containing actinobacteria^52^.

An extensive literature search of phenotypic data acquired on type strains representing each of the species-groups did not reveal any characteristics that could be unambiguously weighted to distinguish between them, a problem compounded by the fact that most validly published rhodococcal species are based on the descriptions of single strains^22^. Previously, we have noted that few standard chemotaxonomic characteristics are available to distinguish *Rhodococcus* strains from other genera classified in the family *Nocardiaceae*, such as *Nocardia* and *Smaragdicoccus*^25^. It can, therefore, be concluded that the taxogenomic approaches employed here reveal stable clustering of representative rhodococci that could not be gleamed using traditional taxonomic criteria. Even so, it is interesting to note that none of the group A *Prescottella* strains, including additional isolates previously investigated^53^,^54^, use L-arabinose, cellobiose, maltose, mannitol, sorbitol and trehalose as sole carbon sources, features shared only with the type strain of *R. triatomae* (a representative of Group G).

In essence, the phylogenetic and taxogenomic data show that strains assigned to rhodococcal species-groups are under-speciated and that many have been misclassified, results that highlight problems associated with the use of current polyphasic approaches to resolve relationships between closely related taxa. These findings are of theoretical value as they provide an insight into matrices that can be used to define generic and species boundaries. The outcomes of this study are also of practical value as they provide a sound basis for improving the classification and identification of rhodococci of agricultural, industrial and medical/veterinary significance, as exemplified by strains assigned to the *R. equi, R. erythropolis* and *R. fascians* species-groups. Importantly, this case study provides tangible evidence that step changes can be made in prokaryotic systematics by “embracing the genome”. Further, it can be anticipated that phylogenetic and taxogenomic procedures will revolutionise the classification and identification of other taxonomically complex actinobacterial taxa, notably the genus *Streptomyces*. Indeed, genome based classification of prokaryotes are likely to become the norm as increasing numbers of whole genomes become available, especially through co-ordinated projects, notably the Genome Encyclopaedia of Bacteria and Archea (GEBA; http://jgi.doe.gov/our-science/science-programs/microbial-genomics/phylogenetic-diversity/).

## Methods

### Bacterial strains and genome sequencing

Fifteen strains: *“Corynebacterium hoagii*”*/R. hoagii* DSM 20295^T^, *R. corynebacterioides* DSM 20151^T^, *R. equi* N1288, N1295 and N1301, *R. gordoniae* DSM 44689^T^, *R. kroppenstedtii* DSM 44908^T^, *R. kunmingensis* DSM 45001^T^, *R. opacus* DSM 43205^T^, *R. phenolicus* DSM 44812^T^, *R. pyridinivorans* DSM 44555^T^, *R. qingshengii* JCM 15477^T^, *R. rhodocorous* DSM 43241^T^, *R. ruber* DSM 43338^T^ and *Rhodococcus* strain AJR001were cultured in 5 ml Brain-Heart Infusion broth (Oxoid) at 28 °C for 48 hours. Genomic DNA was extracted from 1.5 ml culture of each of the strain using an UltraClean^®^ Microbial DNA Isolation Kit (MoBio).

The genome sequencing of *R. kunmingensis* DSM 45001^T^, *R. equi* strains N1288, N1295 and N1301were performed on a Roche GS Junior instrument and reads were assembled into contigs using the GS *de novo* assembler (Roche) and previously defined criteria^34^. The remaining genomes were sequenced on an Illumina MiSeq instrument and the reads were assembled using the CLC Genomic Workbench (Qiagen), as previously defined^33^. The whole genome shotgun sequences of all the strains have been deposited at DDBJ/EMBL/GenBank, the accession numbers are provided in the Supplementary Table 1.

The genome sequences of *R. equi* C7^T^ and *R. defluvii* Ca11^T^ that we have previously sequenced were also included in the analyses^33^,^34^. We also obtained 59 genome sequences of 14 rhodococcal species and 25 genomes of unclassified rhodococci from GenBank (Supplementary Table 1). Representative strains of the genera *Gordonia, Nocardia, Segniliparus, Smardigococcus, Tomitella* and *Williamsia* were also included together with two *C. diphtheriae* genomes^55^,^56^ as an outgroup (Supplementary Table 1).

### Computational analyses

A BLAST-based pairwise average nucleotide identity (ANIb) was calculated from the nucleotide sequences using Jspecies^57^. A matrix of whole genome BLAST-based similarity scores was generated using GEGENEES^45^ using the fast algorithm with a BLAST fragment size of 500 bp. All 115 genome sequences were annotated using the RAST pipeline^35^ to give an equivalence of annotation for the comparative genomic analyses. A subset of amino acids from 400 broadly conserved proteins in prokaryotes was extracted for phylogenetic reconstruction using PhyloPhlAn^38^ with modified MUSCLE^58^ section to compute 16 iterations for refinement of multiple sequence alignment. The best fit substitution model was selected for the final alignment of 3,797 amino acids (VT + F + G4) and a maximum likelihood (ML) tree was generated with 1,000 SH-aLRT (SH-like approximate likelihood ratio test) and ultrafast bootstrap iterations using IQ-Tree^59^,^60^.

The annotated genome sequences were compared using EDGAR^36^ to calculate the core genome and the number of genes shared within each phylogenetic group. For a more comprehensive phylogenetic reconstruction, the nucleotide sequences of 255 core genes were concatenated after removing start and stop codons. A codon based alignment was performed on the concatenated sequence using MUSCLE^58^ in MEGA^61^ with 2 iterations due to computational constraints. The codons with the missing data were striped and a ML tree was generated using the best fit codon substitution model (SCHN05 + F + I + G4) with 1,000 SH-aLRT and ultrafast bootstrap replicates using IQ-Tree^59^,^60^. Another ML tree was constructed using the LG + F + I + G4 amino acid substitution model and 10,000 SH-aLRT and ultrafast bootstrap iterations^59^,^60^ from a concatenated protein sequence alignment of the core genes after removing the sites with missing data and poorly aligned regions using GBLOCKS^62^.

16S rRNA sequences were extracted from 107 of the 115 genomes where the size of the annotated gene was ≥1000 bp. The sequences were aligned using MUSCLE^58^ and the gaps were removed using GBLOCKS^62^, resulting in 641 most reliable sites in the final alignment. A ML tree was constructed using the GTR + I + G4 model with 10,000 SH-aLRT and ultrafast bootstrap iterations using IQ-Tree^59^,^60^. All phylogenetic trees were visualized using the web based program, Interactive Tree Of Life (iTOL)^63^.

The digital DNA-DNA hybridization values were calculated using GGDC 2.1^39^ between representatives of each of the groups that were identified in the phylogenetic and other genomic analyses. A 3D plot from the GC content, genome sizes and the fraction of shared genes within each rhodococcal group (Supplementary Table 1) was generated using PAST^64^.

## Additional Information

**How to cite this article**: Sangal, V. *et al*. Next-generation systematics: An innovative approach to resolve the structure of complex prokaryotic taxa. *Sci. Rep.* **6**, 38392; doi: 10.1038/srep38392 (2016).

**Publisher’s note:** Springer Nature remains neutral with regard to jurisdictional claims in published maps and institutional affiliations.

## Supplementary Material

Supplementary Material
