# Supplementary material for: Next-generation systematics: An innovative approach to resolve the structure of complex prokaryotic taxa
Source: Sci Rep. 2016 Dec 7;6:38392. doi: 10.1038/srep38392 (PMC5141411; doi:10.1038/srep38392)
Supplement: Supplementary Material [file srep38392-s1.pdf]

Next-generation systematics: An innovative approach to resolve the structure of complex prokaryotic taxa

Vartul Sangal[1]*, Michael Goodfellow[2], Amanda L. Jones[1], Edward C. Schwalbe[1], Jochen Blom[3], Paul A. Hoskisson[4], Iain C. Sutcliffe[1]

*Corresponding author

Vartul Sangal

Faculty of Health and Life Sciences

Northumbria University

Newcastle upon Tyne NE1 8ST, UK

Email: vartul.sangal@northumbria.ac.uk

Authors' affiliations:

[1]Faculty of Health and Life Sciences, Northumbria University, Newcastle upon Tyne NE1 8ST, UK

[2]School of Biology, University of Newcastle, Newcastle upon Tyne NE1 7RU, UK

[3]Heinrich-Buff-Ring 58, Justus-Liebig-Universität, 35392 Gießen, Germany

[4]Strathclyde Institute of Pharmacy and Biomedical Sciences, University of Strathclyde, 161 Cathedral Street, Glasgow G4 0RE, UK

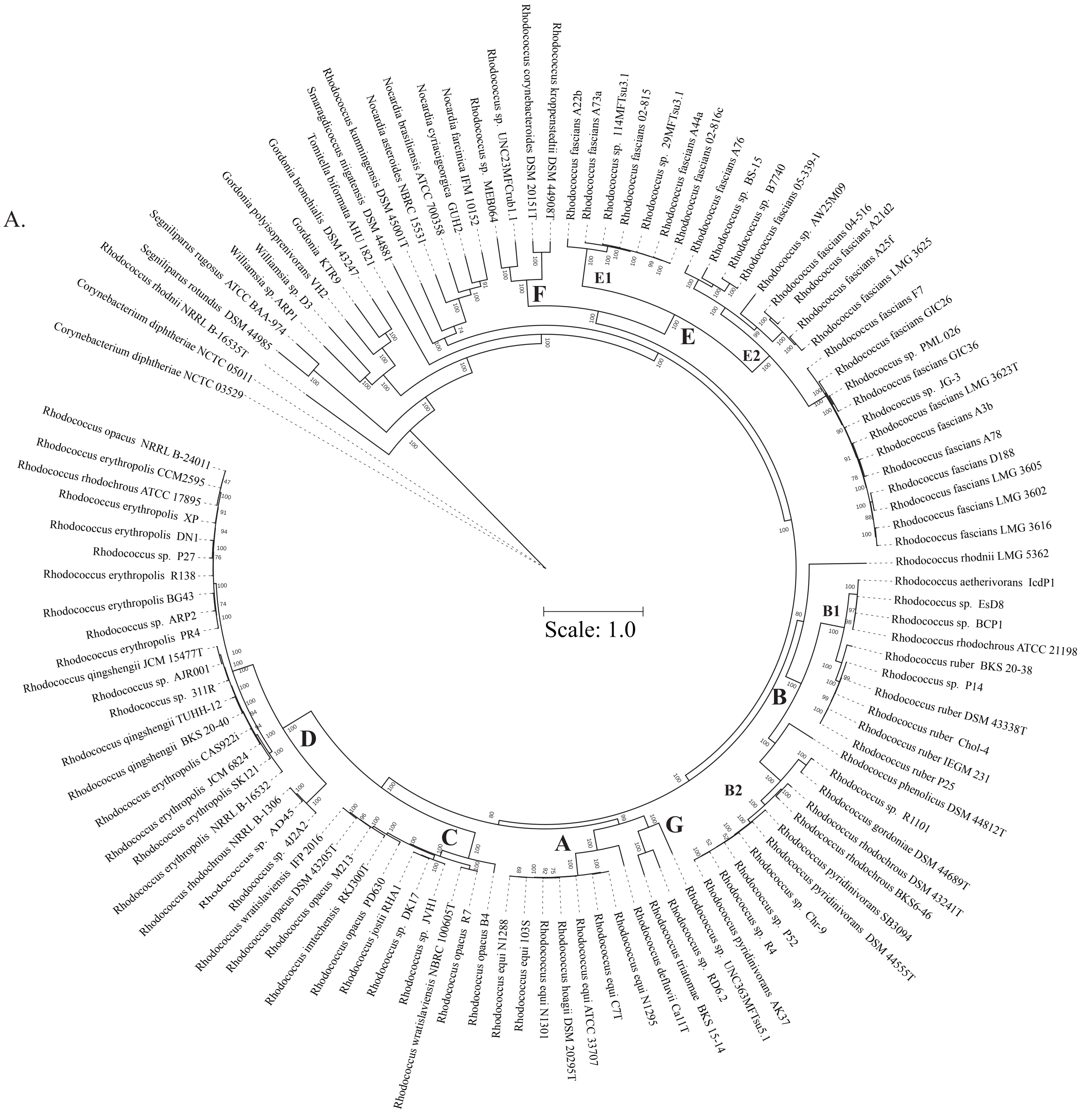

Supplementary Figure 1. The detailed structure of species-groups in the ML tree shown in Fig. 1A. The scale bar represents nucleotide substitutions per codon site.

Supplementary Figure 2. The detailed structure of species-groups in the ML tree shown in Fig. 1B. The scale bar shows normalized fraction of total branch lengths as described by Segata et al. (2013).

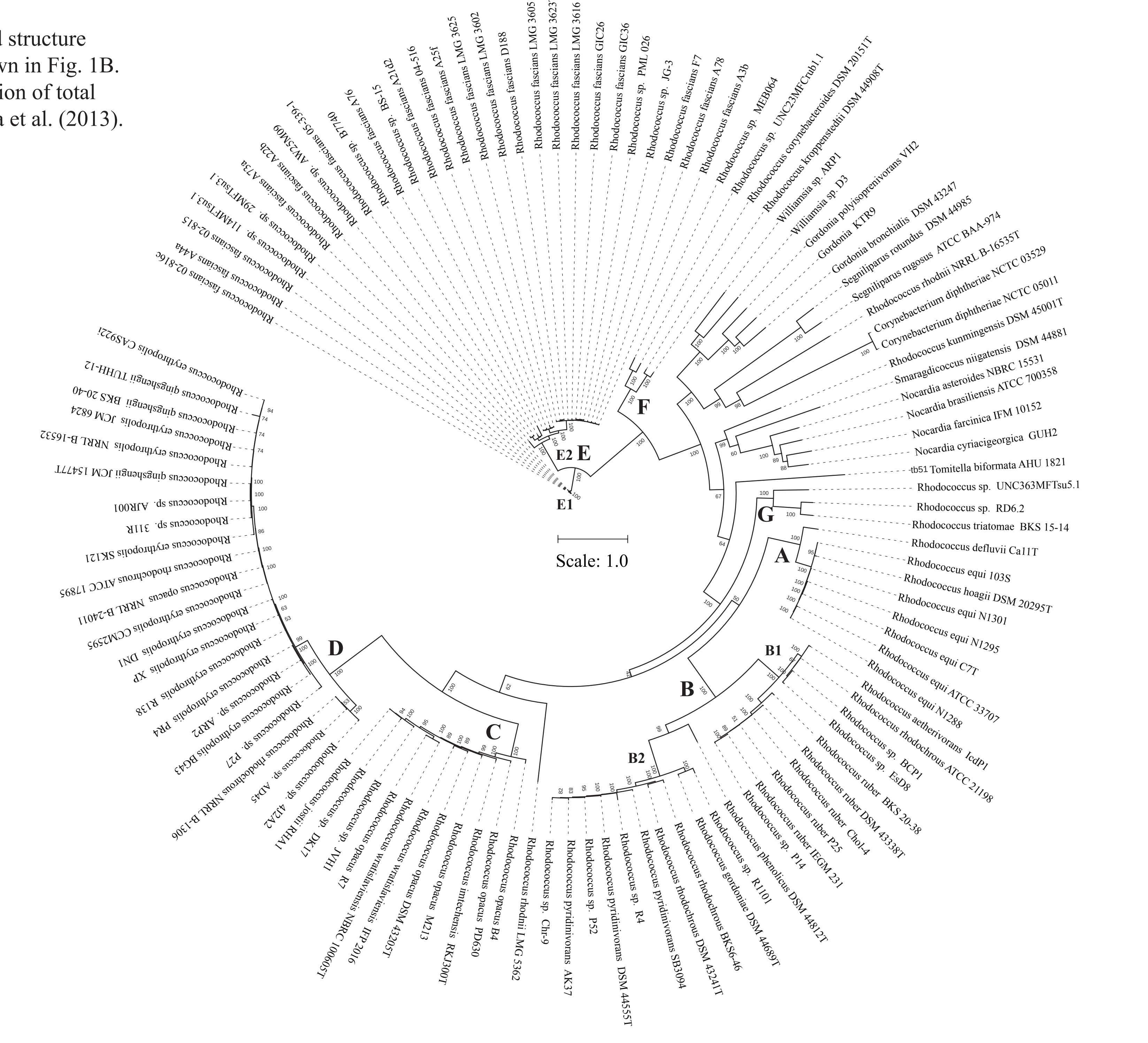

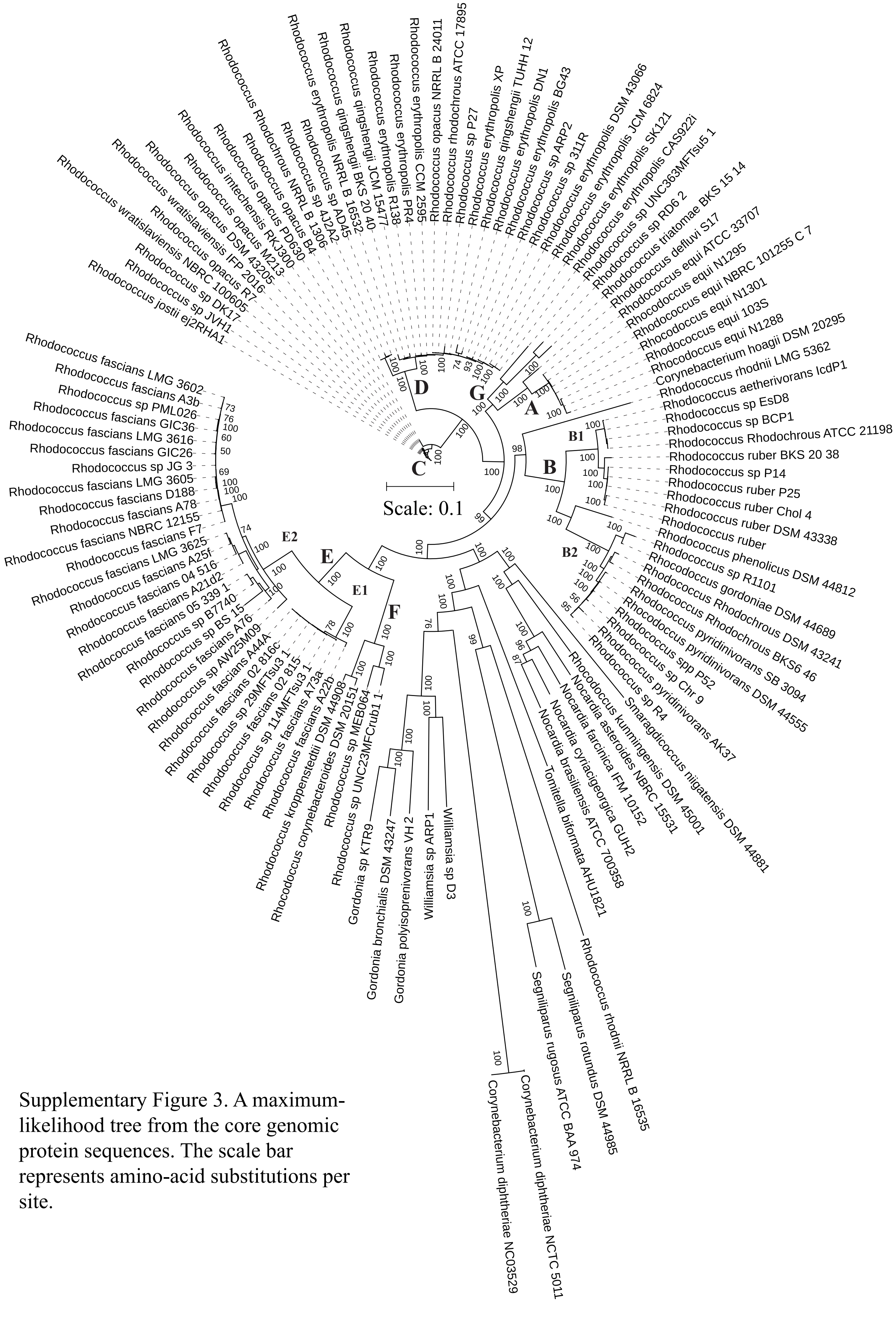

Supplementary Figure 3. A maximum-likelihood tree from the core genomic protein sequences. The scale bar represents amino-acid substitutions per site.

Supplementary Fig. S4. A maximum-likelihood tree from 16S rRNA sequence alignment using GTR+I+G4 Model after removing the sites with the missing data. The scale bar represents nucleotide substitution per site.

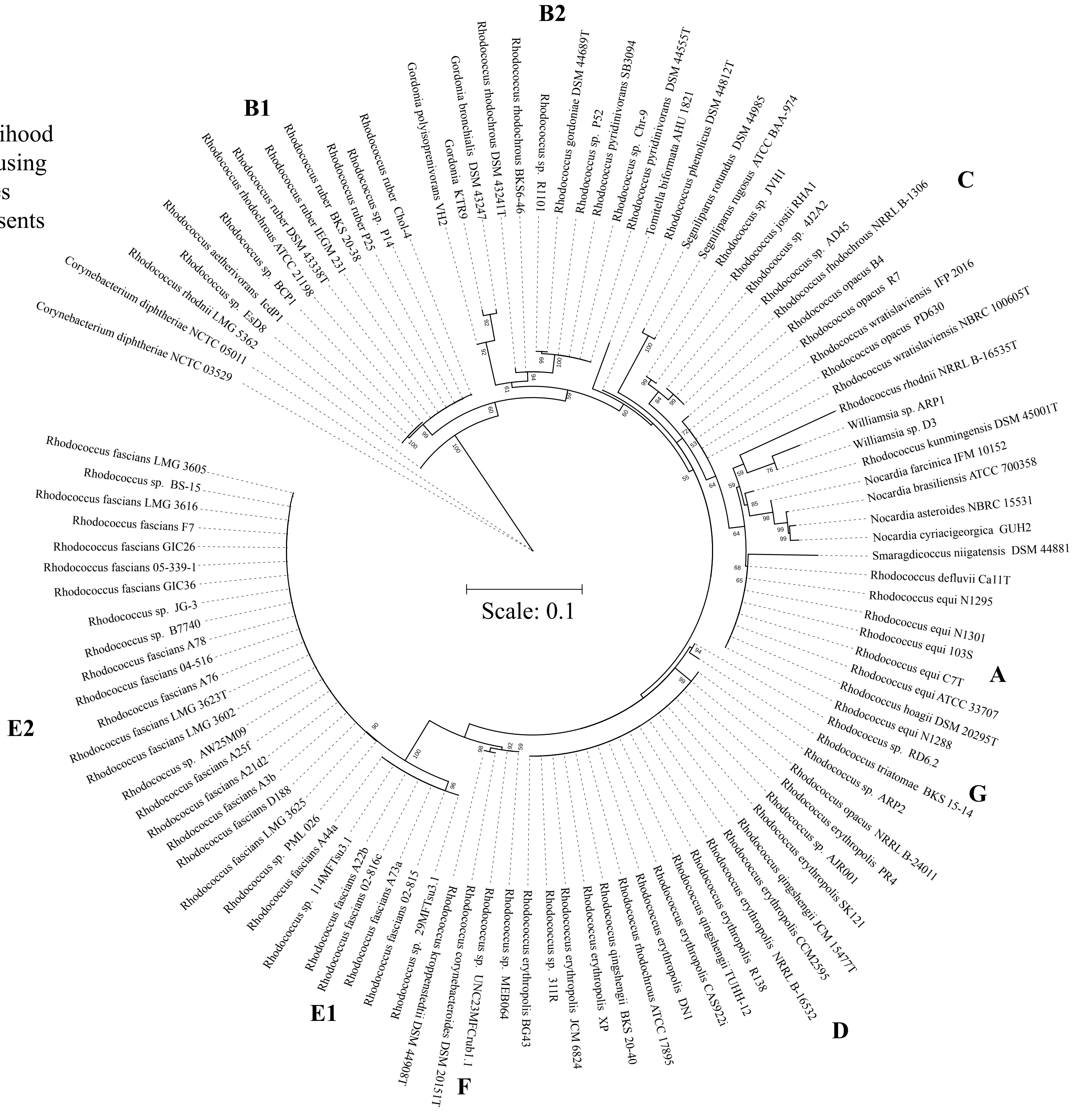

Supplementary Table 1. Details of strains and genomic features

S. no.	S-ID	Organism	Strain	Accession No.	size (Mb)	GC %	#Genes	RNA genes	Group	Species	Fraction
1	re10	Rhodococcus equi	103S	NC_014659	5.04	68.8	4740	64	A	A-1	0.73
2	re11	Rhodococcus equi	N1288	LRQY00000000	5.17	68.8	4942	56	A	A-1	0.70
3	re12	Rhodococcus equi	N1295	LRQZ00000000	5.31	68.7	5078	55	A	A-1	0.68
4	re13	Rhodococcus equi	N1301	LRRA00000000	5.65	68.5	5433	53	A	A-1	0.64
5	re14	Rhodococcus equi	ATCC 33707	NZ_CM001149	5.26	68.8	4986	68	A	A-1	0.69
6	re9	Rhodococcus equi	C7^T	APJC00000000	5.2	68.8	4978	55	A	A-1	0.69
7	ch56	Rhodococcus hoagii	DSM 20295^T	LRRF00000000	4.97	68.8	4705	54	A	A-1	0.73
8	rd55	Rhodococcus defluvii	Ca11^T	JPOC00000000	5.14	68.7	4740	56	A	A-2	0.73
9	r34	Rhodococcus sp.	BCP1	AVAE00000000	6.21	70.4	5889	58	B (B1)	B1-1	0.34
10	r38	Rhodococcus sp.	EsD8	CAVJ000000000	6.63	70.1	6201	51	B (B1)	B1-1	0.32
11	ra97	Rhodococcus aetherivorans	IcdP1	NZ_CP011341.1	5.92	70.6	5589	66	B (B1)	B1-1	0.36
12	rrh31	Rhodococcus rhodochrous	ATCC 21198	AZHI00000000	6.48	70.2	6086	55	B (B1)	B1-1	0.33
13	r42	Rhodococcus sp.	P14	AJFC00000000	5.68	70.4	5253	51	B (B1)	B1-2	0.38
14	rrb102	Rhodococcus ruber	DSM 43338^T	LRRL00000000	5.3	70.7	4934	59	B (B1)	B1-2	0.41
15	rrb103	Rhodococcus ruber	IEGM 231	CCSD01000000	5.99	70.2	5636	55	B (B1)	B1-2	0.35
16	rrb104	Rhodococcus ruber	P25	LDUF01000000	5.73	70.5	5362	58	B (B1)	B1-2	0.37
17	rrb32	Rhodococcus ruber	Chol-4	ANGC00000000	5.4	70.6	5029	67	B (B1)	B1-2	0.40
18	rrb33	Rhodococcus ruber	BKS 20-38	AOEX00000000	6.13	69.7	5758	56	B (B1)	B1-3	0.35
19	rph87	Rhodococcus phenolicus	DSM 44812^T	LRRH00000000	6.28	68.4	6000	58	B	B-4	0.33
20	r41	Rhodococcus sp.	R1101	AJVB00000000	4.66	67.9	4384	58	B (B2)	B2-5	0.46
21	rg82	Rhodococcus gordoniae	DSM 44689^T	LPZN00000000	4.82	67.9	4519	59	B (B2)	B2-5	0.44
22	rrh101	Rhodococcus rhodochrous	DSM 43241^T	LRRK00000000	5.18	68.2	4843	59	B (B2)	B2-6	0.41
23	rrh29	Rhodococcus rhodochrous	BKS6-46	AGVW00000000	6.22	67.4	5612	59	B (B2)	B2-6	0.36
24	r111	Rhodococcus sp.	Chr-9	JTIZ01000000	5.35	67.7	5169	56	B (B2)	B2-7	0.39
25	r37	Rhodococcus sp.	R4	AFAQ00000000	8.86	69.5	8252	109	B (B2)	B2-7	0.24
26	r93	Rhodococcus sp.	P52	JPJJ01000000	5.29	67.9	5061	57	B (B2)	B2-7	0.40
27	rp25	Rhodococcus pyridinivorans	SB3094	NC_023150	5.23	68.0	4956	47	B (B2)	B2-7	0.40
28	rp26	Rhodococcus pyridinivorans	AK37	AHBW00000000	5.25	67.9	5139	55	B (B2)	B2-7	0.39
29	rp88	Rhodococcus pyridinivorans	DSM 44555^T	LRRI00000000	5.18	67.9	4931	56	B (B2)	B2-7	0.41

30	r43	Rhodococcus sp.	JVH1	AKKP00000000	9.18	67.0	8872	55 C	C-1	0.46
31	r44	Rhodococcus sp.	DK17	AJLQ00000000	9.11	67.1	8909	56 C	C-1	0.45
32	rj52	Rhodococcus jostii	RHA1	NC_008268	7.8	67.5	7474	62 C	C-1	0.54
33	ri20	Rhodococcus imtechensis	RKJ300[T]	AJJH00000000	8.23	67.2	8331	54 C	C-2	0.48
34	ro24	Rhodococcus opacus	M213	AJYC00000000	9.2	67.0	8934	57 C	C-2	0.45
35	ro86	Rhodococcus opacus	DSM 43205[T]	LRRG00000000	8.53	67.3	8267	53 C	C-2	0.49
36	rw47	Rhodococcus wratislaviensis	IFP 2016	ANIU00000000	9.7	66.9	9763	64 C	C-2	0.41
39	ro23	Rhodococcus opacus	PD630	AGVD00000000	9.16	67.0	8540	58 C	C-2	0.47
37	ro85	Rhodococcus opacus	R7	CP008947.1	8.47	67.2	7859	60 C	C-3	0.51
38	rw96	Rhodococcus wratislaviensis	NBRC 100605[T]	BAWF01000000	10.41	66.8	9785	57 C	C-3	0.41
40	ro22	Rhodococcus opacus	B4	NC_012522	7.91	67.9	7397	61 C	C-4	0.55
41	r105	Rhodococcus sp.	311R	CFHW01000000	6.35	62.6	6088	57 D	D-1	0.59
42	rey16	Rhodococcus erythropolis	SK121	ACNO00000000	6.79	62.5	6573	56 D	D-1	0.54
43	rey57	Rhodococcus erythropolis	JCM 6824	BBLL01000000	7.02	62.3	6816	54 D	D-1	0.52
44	rey58	Rhodococcus erythropolis	NRRL B-16532	JOIL01000000	6.94	62.4	6746	61 D	D-1	0.53
45	r60	Rhodococcus sp.	AJR001	LPZM00000000	7.26	62.4	7124	56 D	D-1	0.50
46	rey98	Rhodococcus erythropolis	CAS922i	LDPN01000000	7.21	62.3	7001	66 D	D-1	0.51
47	rq27	Rhodococcus qingshengii	BKS 20-40	AODN00000000	6.6	62.4	6431	55 D	D-1	0.55
48	rq89	Rhodococcus qingshengii	TUHH-12	JNCU01000000	7.43	61.7	8298	65 D	D-1	0.43
49	rq90	Rhodococcus qingshengii	JCM 15477[T]	LRRJ00000000	7.26	62.4	7130	56 D	D-1	0.50
50	r107	Rhodococcus sp.	ARP2	LEKE01000000	6.3	62.4	6051	63 D	D-2	0.59
51	r40	Rhodococcus sp.	P27	AVCO00000000	6.26	62.4	6736	60 D	D-2	0.53
52	rey15	Rhodococcus erythropolis	PR4	NC_012490	6.52	62.3	6250	69 D	D-2	0.57
53	rey17	Rhodococcus erythropolis	CCM2595	NC_022115	6.28	62.5	5935	65 D	D-2	0.60
54	rey18	Rhodococcus erythropolis	XP	AGCF00000000	7.23	62.3	7123	67 D	D-2	0.50
55	rey19	Rhodococcus erythropolis	DN1	AUZK00000000	6.55	62.4	6545	56 D	D-2	0.54
56	rey59	Rhodococcus erythropolis	R138	ASKF01000000	6.77	62.3	6486	65 D	D-2	0.55
57	rey99	Rhodococcus erythropolis	BG43	NZ_CP011295.1	6.33	62.4	6011	72 D	D-2	0.59
58	ro84	Rhodococcus opacus	NRRL B-24011	JOIM01000000	6.38	62.4	6040	60 D	D-2	0.59
59	rrh30	Rhodococcus rhodochrous	ATCC 17895	ASJJ00000000	6.87	62.3	6617	53 D	D-2	0.54
60	r108	Rhodococcus sp.	AD45	JYOP01000000	6.79	61.7	6544	54 D	D-3	0.54
61	r113	Rhodococcus sp.	4J2A2	CEDU01000000	6.45	61.8	6211	55 D	D-3	0.57

62	rrh92	Rhodococcus rhodochrous	NRRL B-1306	JNWS01000000	6.78	61.7	6586	59 D	D-3	0.54
63	rf61	Rhodococcus fascians	A22b	JOKB01000000	5.91	64.3	5678	58 E (E1)	E1-1	0.45
64	r35	Rhodococcus sp.	114MFTsu3.1	ARTN00000000	5.55	64.7	5307	56 E (E1)	E1-2	0.48
65	r36	Rhodococcus sp.	29MFTsu3.1	ARND00000000	5.58	64.6	5352	56 E (E1)	E1-2	0.47
66	rf64	Rhodococcus fascians	A44a	JMEX01000000	5.95	64.5	5683	49 E (E1)	E1-2	0.45
67	rf65	Rhodococcus fascians	A73a	JMEW01000000	5.93	64.4	5639	55 E (E1)	E1-2	0.45
68	rf69	Rhodococcus fascians	02-815	JMFF01000000	6.24	64.3	5969	53 E (E1)	E1-2	0.42
69	rf70	Rhodococcus fascians	02-816c	JMFE01000000	6.08	64.5	5956	55 E (E1)	E1-2	0.43
70	rf75	Rhodococcus fascians	A76	JMEV01000000	6.03	64.6	5889	53 E (E2)	E2-3	0.43
71	r110	Rhodococcus sp.	BS-15	BAOX01000000	5.51	64.4	6162	43 E (E2)	E2-4	0.41
72	r114	Rhodococcus sp.	B7740	NZ_CP010797.1	5.34	64.9	5149	59 E (E2)	E2-5	0.49
73	rf72	Rhodococcus fascians	05-339-1	JMFC01000000	5.73	64.7	5561	51 E (E2)	E2-5	0.46
74	rf63	Rhodococcus fascians	04-516	JMFD01000000	5.82	64.2	5588	53 E (E2)	E2-6	0.45
75	rf73	Rhodococcus fascians	A21d2	JMFA01000000	5.98	64.1	5748	57 E (E2)	E2-6	0.44
76	rf74	Rhodococcus fascians	A25f	JMEZ01000000	5.87	64.1	5660	55 E (E2)	E2-6	0.45
77	rf81	Rhodococcus fascians	LMG 3625	JMEM01000000	5.94	64.1	5886	58 E (E2)	E2-6	0.43
78	r112	Rhodococcus sp.	PML 026	JZIS01000000	5.17	64.7	4965	60 E (E2)	E2-7	0.51
79	r39	Rhodococcus sp.	JG-3	AXVF00000000	5.27	64.6	5100	54 E (E2)	E2-7	0.50
80	rf100	Rhodococcus fascians	F7	LFDS01000000	5.25	64.7	5047	50 E (E2)	E2-7	0.50
81	rf62	Rhodococcus fascians	A3b	JMEY01000000	6.03	64.2	5846	55 E (E2)	E2-7	0.43
82	rf66	Rhodococcus fascians	A78	JMEU01000000	6	64.3	5783	53 E (E2)	E2-7	0.44
83	rf67	Rhodococcus fascians	GIC26	JMES01000000	5.34	64.5	5176	50 E (E2)	E2-7	0.49
84	rf68	Rhodococcus fascians	GIC36	JMER01000000	5.56	64.5	5392	52 E (E2)	E2-7	0.47
85	rf76	Rhodococcus fascians	D188	JMET01000000	5.44	64.6	5222	50 E (E2)	E2-7	0.48
86	rf77	Rhodococcus fascians	LMG 3602	JMEQ01000000	5.36	64.5	5227	53 E (E2)	E2-7	0.48
87	rf78	Rhodococcus fascians	LMG 3605	JMEP01000000	5.44	64.5	5215	52 E (E2)	E2-7	0.49
88	rf79	Rhodococcus fascians	LMG 3616	JMEO01000000	5.76	64.3	5567	52 E (E2)	E2-7	0.45
89	rf80	Rhodococcus fascians	LMG 3623^T	JMEN01000000	5.78	64.4	5519	52 E (E2)	E2-7	0.46
90	r45	Rhodococcus sp.	AW25M09	CAPS00000000	5.64	64.1	5522	50 E (E2)	E2-8	0.46
91	rc54	Rhodococcus corynebacteroides	DSM 20151^T	LPZL00000000	3.9	70.3	3607	50 F	F-1	0.75
92	rkr83	Rhodococcus kroppenstedtii	DSM 44908^T	LPZO00000000	3.91	70.1	3650	47 F	F-2	0.74
93	r94	Rhodococcus sp.	UNC23MFCrub1.1	JMLQ01000000	4.7	68.5	4440	55 F	F-3	0.61
94	r109	Rhodococcus sp.	MEB064	JXQS01000000	4.67	67.0	4415	52 F	F-4	0.62

95	r95	Rhodococcus sp.	UNC363MFTsu5.1	JMLO01000000	5.67	69.3	5300	57	G	G-1	0.61
96	r106	Rhodococcus sp.	RD6.2	CVQP01000000	5.57	68.4	5216	64	G	G-2	0.62
97	rt46	Rhodococcus triatomae	BKS 15-14	AODO00000000	5.83	69.0	5416	57	G	G-3	0.59
98	rk21	Rhodococcus kunmingensis	DSM 45001^{T}	LRRB00000000	5.62	66.2	5234	50	-	-	
99	rr28	Rhodococcus rhodnii	LMG 5362	APMY00000000	4.39	69.7	4251	54	-	-	
100	rr91	Rhodococcus rhodnii	NRRL B-16535^{T}	JOAA01000000	12.42	66.3	11568	79	-	-	
101	na53	Nocardia asteroides	NBRC 15531	BAFO02000000	6.96	69.9	6425	56	-	-	
102	nb6	Nocardia brasiliensis	ATCC 700358	NC_018681	9.44	68.1	8769	58	-	-	
103	nc7	Nocardia cyriacigeorgica	GUH2	NC_016887	6.19	68.4	5663	58	-	-	
104	nf8	Nocardia farcinica	IFM 10152	NC_006361	6.02	70.8	5738	62	-	-	
105	g3	Gordonia	KTR9	NC_018581	5.44	67.8	4985	54	-	-	
106	gb4	Gordonia bronchialis	DSM 43247	NC_013441	5.21	67.1	5038	53	-	-	
107	gp5	Gordonia polyisoprenivorans	VH2	NC_016906	5.67	67.0	5169	58	-	-	
108	sr48	Segniliparus rotundus	DSM 44985	NC_014168	3.16	66.8	3150	49	-	-	
109	srg49	Segniliparus rugosus	ATCC BAA-974	ACZI00000000	3.57	68.1	3535	49	-	-	
110	smn50	Smaragdicoccus niigatensis	DSM 44881	AQXZ00000000	5.32	64.3	5234	52	-	-	
111	tb51	Tomitella biformata	AHU 1821	BAVQ00000000	4.71	68.1	4389	56	-	-	
112	w115	Williamsia sp.	ARP1	JXYP00000000	4.75	68.6	4460	52	-	-	
113	w116	Williamsia sp.	D3	AYTE00000000	5.62	64.6	5632	51	-	-	
114	cd1	Corynebacterium diphtheriae	NCTC 03529	AJGI00000000	2.48	53.6	2490	56	-	-	
115	cd2	Corynebacterium diphtheriae	NCTC 05011	AJVH00000000	2.38	53.6	2341	51	-	-	

Notes:
1. The strains sequenced in this study are shown in red.
2. The subgroup information is provided in parenthesis in the column "Group".
3. The predicted species from the genomic analyses are coded with numbers following the group/subgroup designation.
4. The fraction of the genes in the core genome within the group is mentioned in the column "Fraction".

Supplementary Table 2a. Pairwise fragmented BLAST similarity scores from the genome sequences

	ch56b	re10	re11b	re12b	re13b	re14	re9	rd55	r34	r38	ra97	rrh31	r42	rrb102b	rrb103	rrb104	rrb32	rrb33	rph87b	r41	rg82b	rrh101b	rrh29	r111	r37	r93	rp25	rp26	rp88b	r43	r44	rj52	ri20	ro24	ro86b	rw47
ch56		93.01	94.80	92.56	94.08	92.20	92.98	31.30	9.53	9.49	9.59	9.50	8.99	9.00	9.05	9.02	9.03	8.67	9.43	7.30	7.21	8.04	8.18	7.53	7.55	7.41	7.87	7.46	7.41	9.55	9.29	9.52	8.69	9.48	9.52	9.47
re10	91.56		94.38	92.66	93.99	93.97	93.00	31.90	9.91	9.80	10.00	9.87	9.19	9.25	9.38	9.29	9.27	8.78	9.21	7.63	7.68	8.14	8.41	7.80	7.73	7.72	8.13	7.72	7.74	9.67	9.23	9.71	8.73	9.70	9.65	9.59
re11	90.87	91.91		92.37	93.39	91.87	93.00	30.78	9.43	9.29	9.45	9.35	8.82	8.88	8.92	8.90	8.89	8.56	9.14	7.27	7.19	7.82	8.08	7.42	7.45	7.29	7.73	7.36	7.33	9.28	9.06	9.28	8.45	9.17	9.16	9.09
re12	86.56	88.03	90.08		88.92	90.53	96.78	30.15	9.27	9.12	9.23	9.22	8.60	8.63	8.70	8.68	8.69	8.29	8.56	7.02	6.98	7.59	7.75	7.17	7.25	7.10	7.51	7.19	7.15	9.02	8.76	8.98	8.23	9.02	9.01	8.97
re13	82.61	83.84	85.54	83.49		84.16	83.65	28.49	9.55	9.41	9.62	9.53	8.88	8.92	9.00	8.99	8.97	8.72	8.53	6.60	6.57	7.17	7.59	6.80	6.86	6.71	7.08	6.83	6.76	8.54	8.39	8.51	7.70	8.37	8.38	8.29
re14	87.36	90.45	90.80	91.74	90.70		91.92	30.84	9.66	9.56	9.71	9.63	8.91	9.00	9.03	8.99	8.98	8.63	8.97	7.44	7.37	7.98	8.18	7.54	7.45	7.44	7.89	7.47	7.48	9.49	9.04	9.53	8.50	9.28	9.39	9.36
re9	88.72	90.13	92.58	98.77	90.92	92.55		30.73	9.43	9.36	9.46	9.39	8.68	8.77	8.80	8.77	8.77	8.43	8.75	7.14	7.12	7.94	8.00	7.37	7.44	7.24	7.72	7.37	7.29	9.17	8.91	9.16	8.45	9.22	9.18	9.13
rd55	30.51	31.19	31.11	31.27	31.32	31.37	31.37		9.74	9.62	9.81	9.70	9.02	8.99	9.10	9.07	9.04	8.78	8.91	7.05	7.30	7.94	8.13	7.71	7.77	7.59	7.58	7.71	7.67	9.44	9.27	9.46	8.69	9.38	9.37	9.32
r34	7.83	7.98	8.01	8.07	8.86	8.04	8.06	8.12		88.38	84.62	92.22	57.52	55.52	57.33	57.29	55.76	56.07	14.64	10.64	11.69	11.86	12.78	12.12	11.99	12.55	11.38	12.15	12.60	9.46	9.36	9.32	8.90	9.51	9.31	9.84
r38	7.23	7.35	7.36	7.39	8.12	7.39	7.38	7.46	83.12		80.64	82.22	55.21	53.10	57.06	56.50	53.58	53.55	13.73	10.04	10.93	11.05	12.09	11.25	11.45	11.91	10.68	11.84	11.87	8.81	9.10	8.64	8.04	8.56	8.77	9.00
ra97	8.35	8.54	8.47	8.50	9.39	8.50	8.51	8.68	88.84	90.33		89.66	60.16	58.92	60.58	60.35	59.27	59.15	14.94	11.44	11.90	12.48	13.59	12.14	12.23	12.20	12.20	12.40	12.30	9.47	9.52	9.62	8.65	9.41	9.29	9.59
rrh31	7.37	7.49	7.51	7.61	8.36	7.56	7.56	7.62	88.60	83.99	81.70		54.82	52.61	56.82	56.48	54.61	54.76	14.03	10.17	11.28	11.39	12.29	11.73	11.69	12.36	10.94	12.12	12.24	8.69	8.85	8.85	7.92	8.62	8.52	8.84
r42	7.92	8.06	8.09	8.11	8.91	8.15	8.08	8.19	63.27	64.56	63.07	62.64		87.06	89.51	88.00	87.09	72.18	14.32	11.00	11.60	11.96	12.89	12.22	12.58	12.49	11.78	12.72	12.37	8.79	8.74	8.51	8.15	8.64	8.93	9.12
rrb102	8.36	8.49	8.54	8.55	9.43	8.60	8.55	8.71	64.87	66.21	65.62	64.18	92.43		91.95	92.15	93.77	74.89	15.14	11.67	12.21	12.71	13.70	12.78	12.83	12.87	12.54	13.16	12.67	9.13	9.15	8.89	8.45	8.94	9.16	9.19
rrb103	7.72	7.92	7.86	7.90	8.75	7.87	7.87	7.96	60.02	63.37	60.13	61.73	84.83	82.00		88.80	84.17	69.39	14.23	10.78	11.62	11.78	12.71	12.41	12.53	12.87	11.75	13.37	12.75	8.49	8.46	8.38	7.84	8.61	8.74	9.01
rrb104	7.83	8.02	8.01	8.04	8.90	8.04	8.02	8.17	62.08	64.94	62.21	63.38	86.51	85.39	91.89		88.48	72.52	14.65	11.01	12.36	12.14	13.15	12.66	12.66	13.46	12.00	13.31	13.16	8.50	8.62	8.59	8.11	8.85	8.60	9.18
rrb32	8.32	8.50	8.52	8.57	9.48	8.56	8.54	8.60	63.85	65.19	64.68	64.92	90.83	92.12	92.52	93.69		74.87	15.36	11.72	12.46	12.78	13.67	13.00	13.18	13.39	12.54	13.78	13.24	8.95	8.98	9.01	8.52	9.27	8.94	9.36
rrb33	7.13	7.25	7.26	7.29	8.11	7.27	7.27	7.38	56.58	57.54	56.97	57.50	66.22	64.75	67.28	67.73	65.93		12.94	9.90	10.75	11.08	11.91	11.29	11.23	11.69	10.60	11.31	11.48	8.03	8.15	8.05	7.56	8.11	8.28	8.60

Supplementary Table 2b. Pairwise ANI-G score from the genome sequences

	ch56	re13	re12	re9	re14	re10	re11	rd55	r34	rrh31	r38	ra97	r42	rrb103	rrb102	rrb32	rrb104	rrb33	rph87	rg82	r41	rrh101	rrh29	r37	r93	rp88	rp25	rp26	r111	r44	rj52	r43	ro24	ri20	rw47	ro86	ro23	ro85	rw96	ro22	rey16	rq27	rey98	rey57	rq89	rey58	rq90	r60	r105	rey18	rey59	r107	r40	rey19	rey17	rey15	ro84	rey99	rrh30	r113	r108	rrh92	rf61	rf70	r36	r35	rf64	rf69	rf65	rf75	r110	rf72	r114	rf73	rf81	rf63	rf74	rf78	r112	rf77	rf76	rf80	rf66	rf79	rf62	rf67	rf68	rf100	r39	r45	rc54	rkr83	r94	r109	r95	r106	rt46	rk21	rr28	rr91	na53	nb6	nc7	nf8	g3	gb4	gp5	cd1	cd2	w115	w116	sr48	srg49	smn50	tb51
ch56	---	99.15	98.84	98.81	98.76	98.99	99.11	83.15	75.25	75.23	75.22	75.25	75.17	75.10	75.16	75.12	75.14	74.87	74.85	74.08	74.29	74.33	74.30	74.03	74.14	74.15	74.36	74.25	74.18	75.37	75.46	75.41	75.48	75.33	75.51	75.52	75.59	75.53	75.42	75.61	73.13	73.12	73.13	73.16	73.15	73.19	73.14	73.12	73.27	73.08	73.17	73.16	72.93	73.16	73.22	73.17	73.21	73.19	73.19	72.72	72.69	72.69	72.40	72.41	72.46	72.37	72.43	72.43	72.40	72.70	72.82	72.80	72.83	72.39	72.31	72.30	72.35	72.59	72.54	72.58	72.59	72.66	72.65	72.63	72.63	72.56	72.50	72.55	72.63	72.27	72.91	72.92	72.74	72.36	75.92	75.19	75.27	72.77	###	###	73.09	72.60	73.01	73.24	70.88	###	###	66.25	66.42	70.85	70.39	69.20	69.85	71.00	####
re13	98.92	---	98.55	98.68	98.55	98.87	98.96	83.07	75.47	75.37	75.42	75.56	75.24	75.22	75.28	75.27	75.23	75.08	74.68	73.88	74.04	73.97	74.23	73.76	73.91	73.88	74.20	74.03	73.92	74.98	75.16	75.04	74.98	74.95	75.07	75.09	75.12	75.15	75.04	75.16	73.05	73.01	72.99	73.06	72.97	73.02	73.00	73.00	73.10	72.90	73.01	73.00	72.77	73.02	73.09	73.02	73.08	73.02	73.02	72.59	72.63	72.65	72.18	72.25	72.29	72.29	72.22	72.28	72.19	72.37	72.47	72.50	72.58	72.18	72.14	72.23	72.08	72.33	72.32	72.30	72.32	72.38	72.32	72.33	72.26	72.30	72.19	72.39	72.40	71.92	72.58	72.57	72.40	71.94	75.73	75.05	75.26	72.45	###	###	72.89	72.43	72.74	73.04	70.55	###	###	66.10	66.25	70.71	70.03	69.36	69.71	70.87	####
re12	98.61	98.61	---	99.97	98.77	98.62	98.85	83.15	75.21	75.15	75.18	75.19	75.00	74.91	75.04	74.94	74.92	74.81	74.71	73.96	74.07	74.12	74.13	73.85	73.95	73.93	74.23	74.05	74.04	75.08	75.30	75.16	75.33	75.19	75.42	75.32	75.39	75.33	75.27	75.46	73.09	73.01	73.01	73.12	73.03	73.08	72.97	72.97	73.11	72.99	73.04	73.00	72.84	73.04	73.11	73.05	73.12	73.09	73.09	72.65	72.65	72.67	72.09	72.21	72.19	72.18	72.25	72.22	72.20	72.37	72.59	72.55	72.63	72.20	72.05	72.19	72.14	72.25	72.26	72.27	72.25	72.26	72.29	72.27	72.22	72.25	72.24	72.26	72.31	72.08	72.63	72.65	72.50	72.02	75.74	75.09	75.26	72.64	###	###	72.75	72.53	72.88	73.14	70.55	###	###	66.24	66.23	70.72	70.03	69.06	69.65	70.76	####
re9	98.57	98.71	99.98	---	98.81	98.60	98.82	83.11	75.20	75.15	75.15	75.23	75.10	75.06	75.04	75.03	75.06	74.94	74.64	74.06	74.15	74.10	74.06	73.89	74.00	74.00	74.24	74.15	74.03	75.12	75.24	75.25	75.26	75.06	75.31	75.32	75.34	75.27	75.18	75.41	73.15	73.10	73.08	73.17	73.12	73.16	73.06	73.08	73.21	73.05	73.07	73.11	72.91	73.12	73.15	73.10	73.17	73.17	73.14	72.75	72.74	72.78	72.25	72.22	72.25	72.21	72.28	72.25	72.28	72.64	72.75	72.67	72.77	72.38	72.25	72.33	72.37	72.53	72.45	72.51	72.52	72.55	72.56	72.58	72.46	72.48	72.42	72.51	72.59	72.16	72.71	72.75	72.69	72.07	75.75	75.05	75.17	72.56	###	###	72.91	72.51	73.04	73.27	70.70	###	###	66.46	66.49	70.91	70.18	69.29	70.00	70.98	####
re14	98.56	98.55	98.76	98.81	---	98.83	98.73	83.25	75.28	75.22	75.29	75.28	75.21	75.17	75.21	75.19	75.17	75.03	74.83	74.16	74.45	74.28	74.33	73.97	74.16	74.21	74.46	74.22	74.14	75.10	75.33	75.33	75.40	75.20	75.54	75.50	75.43	75.37	75.37	75.47	73.23	73.20	73.17	73.28	73.24	73.24	73.22	73.23	73.25	73.17	73.15	73.14	72.82	73.21	73.24	73.16	73.24	73.20	73.26	72.84	72.82	72.84	72.23	72.33	72.32	72.32	72.37	72.38	72.34	72.62	72.91	72.72	72.87	72.45	72.25	72.36	72.37	72.40	72.46	72.45	72.40	72.47	72.41	72.51	72.40	72.40	72.29	72.48	72.60	72.19	72.99	72.99	72.72	72.21	75.78	75.26	75.40	72.90	###	###	73.12	72.74	73.20	73.43	70.93	###	###	66.83	66.74	71.22	70.37	69.85	70.30	71.03	####
re10	98.97	99.11	98.81	98.81	99.03	---	99.06	83.24	75.41	75.33	75.36	75.40	75.27	75.21	75.31	75.23	75.16	75.02	74.92	74.27	74.52	74.39	74.51	74.09	74.28	74.28	74.58	74.39	74.26	75.15	75.39	75.38	75.49	75.21	75.51	75.44	75.49	75.43	75.36	75.59	73.19	73.24	73.16	73.28	73.22	73.21	73.27	73.26	73.29	73.25	73.22	73.20	72.91	73.24	73.21	73.19	73.23	73.25	73.23	72.82	72.79	72.78	72.27	72.49	72.37	72.41	72.53	72.47	72.46	72.61	72.79	72.67	72.75	72.38	72.22	72.32	72.29	72.39	72.42	72.43	72.39	72.47	72.43	72.44	72.41	72.30	72.28	72.49	72.53	72.21	72.93	72.95	72.77	72.28	75.83	75.38	75.50	72.84	###	###	73.17	72.66	73.14	73.32	70.87	###	###	66.62	66.69	71.00	70.32	69.38	69.81	70.96	####
re11	99.00	99.06	98.93	98.90	98.80	98.92	---	83.03	75.12	75.10	75.08	75.11	75.08	74.98	75.04	75.04	75.02	74.81	74.82	73.96	74.19	74.07	74.26	73.88	74.01	73.99	74.28	74.10	73.98	75.11	75.26	75.22	75.31	75.12	75.35	75.31	75.38	75.21	75.16	75.30	73.07	73.15	73.05	73.12	73.08	73.10	73.11	73.10	73.14	73.18	73.03	73.03	72.83	73.08	73.09	73.02	73.11	73.06	73.07	72.68	72.65	72.67	72.21	72.30	72.26	72.24	72.30	72.30	72.29	72.63	72.80	72.69	72.78	72.32	72.14	72.33	72.26	72.49	72.39	72.42	72.48	72.48	72.43	72.43	72.42	72.42	72.34	72.44	72.51	72.22	72.84	72.89	72.64	72.19	75.87	75.20	75.31	72.80	###	###	72.96	72.52	72.98	73.11	70.71	###	###	66.30	66.36	70.94	70.20	69.29	69.89	70.80	####
rd55	82.90	82.95	83.03	83.00	83.03	82.96	82.93	---	75.51	75.53	75.49	75.52	75.27	75.32	75.37	75.29	75.31	75.13	75.15	74.15	74.14	74.25	74.34	74.03	74.23	74.29	74.23	74.37	74.29	75.43	75.54	75.42	75.40	75.38	75.53	75.52	75.50	75.40	75.42	75.56	73.31	73.31	73.24	73.45	73.27	73.34	73.30	73.29	73.34	73.24	73.35	73.27	73.10	73.28	73.34	73.27	73.34	73.31	73.37	73.16	73.16	73.18	72.28	72.46	72.42	72.44	72.50	72.50	72.45	72.80	72.93	72.88	72.92	72.49	72.42	72.51	72.46	72.59	72.54	72.61	72.60	72.69	72.65	72.63	72.52	72.57	72.49	72.61	72.68	72.40	73.01	72.87	72.82	72.45	76.28	75.71	75.34	72.85	###	###	73.13	72.69	73.15	73.39	70.88	###	###	66.49	66.51	71.12	70.30	69.27	70.01	70.99	####
r34	75.04	75.43	75.08	75.02	75.07	75.10	75.03	75.31	---	99.34	98.67	98.52	90.93	90.83	90.72	90.66	90.78	90.40	78.51	77.22	76.90	77.04	77.38	76.96	77.46	77.45	77.09	77.27	77.39	75.18	75.32	75.28	75.40	75.27	75.59	75.29	75.34	75.37	75.42	75.29	72.52	72.49	72.55	72.45	72.53	72.52	72.51	72.52	72.51	72.44	72.47	72.53	72.30	72.51	72.59	72.50	72.60	72.58	72.54	71.99	72.10	71.96	71.56	71.79	71.75	71.76	71.75	71.70	71.79	71.99	72.19	72.06	72.17	71.75	71.69	71.70	71.80	71.78	71.91	71.78	71.80	71.88	71.74	71.87	71.75	71.77	71.77	71.87	71.99	71.76	72.79	72.89	72.48	72.02	74.98	74.38	74.49	72.55	###	###	72.94	72.52	73.09	73.44	70.83	###	###	66.69	66.61	71.21	70.10	69.39	70.04	70.33	####
rrh31	75.08	75.41	75.01	75.04	75.06	75.04	75.05	75.29	99.19	---	98.48	98.57	90.91	90.89	90.75	90.80	90.80	90.44	78.54	77.19	77.02	77.22	77.51	77.05	77.50	77.52	77.13	77.48	77.45	75.20	75.38	75.17	75.22	75.14	75.45	75.25	75.25	75.21	75.25	75.27	72.51	72.48	72.50	72.51	72.49	72.49	72.49	72.48	72.52	72.52	72.51	72.57	72.39	72.58	72.55	72.60	72.52	72.61	72.53	72.04	72.10	72.02	71.56	71.82	71.80	71.74	71.70	71.74	71.74	71.92	72.03	71.98	72.10	71.68	71.65	71.64	71.72	71.80	71.72	71.71	71.82	71.72	71.60	71.73	71.66	71.72	71.58	71.74	71.82	71.61	72.79	72.94	72.45	71.86	74.96	74.48	74.66	72.56	###	###	72.88	72.53	73.11	73.52	70.78	###	###	66.29	66.38	70.99	70.07	69.43	69.96	70.37	####
r38	74.96	75.28	75.02	74.98	75.00	74.99	74.93	75.13	98.28	98.21	---	98.75	91.17	91.36	91.03	90.88	91.31	90.47	78.38	77.32	77.17	77.04	77.50	77.12	77.50	77.45	77.19	77.58	77.30	75.20	75.19	75.20	75.22	75.12	75.43	75.28	75.28	75.33	75.30	75.38	72.43	72.41	72.46	72.46	72.45	72.50	72.40	72.41	72.46	72.40	72.37	72.44	72.31	72.44	72.52	72.43	72.50	72.49	72.46	71.88	72.03	71.89	71.47	71.68	71.71	71.63	71.61	71.65	71.67	71.78	72.01	71.79	71.96	71.54	71.67	71.49	71.69	71.72	71.74	71.71	71.71	71.75	71.64	71.67	71.58	71.78	71.68	71.73	71.87	71.58	72.39	72.56	72.28	71.78	74.74	74.13	74.39	72.48	###	###	72.87	72.43	72.92	73.24	70.62	###	###	66.33	66.29	70.89	69.86	69.41	69.95	70.23	####
ra97	[illegible]
r42	[illegible]
rrb103	[illegible]
rrb102	[illegible]
rrb32	[illegible]
rrb104	[illegible]
rrb33	[illegible]
rph87	[illegible]
rg82	[illegible]
r41	[illegible]
rrh101	[illegible]
rrh29	[illegible]
r37	[illegible]
r93	[illegible]
rp88	[illegible]
rp25	[illegible]
rp26	[illegible]
r111	[illegible]
r44	[illegible]
rj52	[illegible]
r43	[illegible]
ro24	[illegible]
ri20	[illegible]
rw47	[illegible]
ro86	[illegible]
ro23	[illegible]
ro85	[illegible]
rw96	[illegible]
ro22	[illegible]
rey16	[illegible]
rq27	[illegible]
rey98	[illegible]
rey57	[illegible]
rq89	[illegible]
rey58	[illegible]
rq90	[illegible]
r60	[illegible]
r105	[illegible]
rey18	[illegible]
rey59	[illegible]
r107	[illegible]
r40	[illegible]
rey19	[illegible]
rey17	[illegible]
rey15	[illegible]
ro84	[illegible]
rey99	[illegible]
rrh30	[illegible]
r113	[illegible]
r108	[illegible]
rrh92	[illegible]
rf61	[illegible]
rf70	[illegible]
r36	[illegible]
r35	[illegible]
rf64	[illegible]
rf69	[illegible]
rf65	[illegible]
rf75	[illegible]
r110	[illegible]
rf72	[illegible]
r114	[illegible]
rf73	[illegible]
rf81	[illegible]
rf63	[illegible]
rf74	[illegible]
rf78	[illegible]
r112	[illegible]
rf77	[illegible]
rf76	[illegible]
rf80	[illegible]
rf66	[illegible]
rf79	[illegible]
rf62	[illegible]
rf67	[illegible]
rf68	[illegible]
rf100	[illegible]
r39	[illegible]
r45	[illegible]
rc54	[illegible]
rkr83	[illegible]
r94	[illegible]
r109	[illegible]
r95	[illegible]
r106	[illegible]
rt46	[illegible]
rk21	[illegible]
rr28	[illegible]
rr91	[illegible]
na53	[illegible]
nb6	[illegible]
nc7	[illegible]
nf8	[illegible]
g3	[illegible]
gb4	[illegible]
gp5	[illegible]
cd1	[illegible]
cd2	[illegible]
w115	[illegible]
w116	[illegible]
sr48	[illegible]
srg49	[illegible]
smn50	[illegible]
tb51	[illegible]

Supplementary Table 2c. Pairwise ANI-C score from 255 core genes

	ch56	re13	re12	re9	re14	re10	re11	rd55	r38	rrh31	r34	ra97	r42	rrb103	rrb102	rrb32	rrb104	rrb33	rph87	rg82
ch56	---	99.7	99.49	99.51	99.49	99.6	99.6	89.3	82.7	82.7	82.7	82.8	82.5	82.49	82.55	82.53	82.5	82.5	82.37	81.5
re13	99.66	---	99.49	99.5	99.47	99.6	99.6	89.3	82.6	82.7	82.7	82.7	82.5	82.47	82.53	82.52	82.5	82.4	82.35	81.6
re12	99.49	99.5	---	99.99	99.5	99.5	99.5	89.3	82.7	82.7	82.7	82.7	82.5	82.44	82.48	82.47	82.5	82.4	82.34	81.6
re9	99.51	99.5	99.99	---	99.51	99.5	99.5	89.3	82.7	82.7	82.7	82.8	82.5	82.46	82.5	82.49	82.5	82.4	82.36	81.6
re14	99.48	99.5	99.5	99.51	---	99.5	99.5	89.3	82.6	82.7	82.7	82.7	82.5	82.49	82.45	82.44	82.5	82.4	82.38	81.5
re10	99.62	99.6	99.5	99.5	99.46	---	99.6	89.3	82.6	82.7	82.7	82.7	82.5	82.41	82.43	82.42	82.4	82.4	82.29	81.5
re11	99.58	99.6	99.48	99.49	99.47	99.6	---	89.3	82.6	82.6	82.7	82.7	82.5	82.41	82.42	82.41	82.4	82.4	82.3	81.6
rd55	89.24	89.3	89.27	89.26	89.24	89.2	89.2	---	82.6	82.7	82.7	82.7	82.5	82.52	82.49	82.47	82.5	82.4	82.37	81.5
r38	82.69	82.7	82.73	82.68	82.66	82.7	82.7	82.7	---	99.8	99.8	99.8	94.9	94.91	94.92	94.94	94.9	94.7	85.96	84.6
rrh31	82.69	82.7	82.73	82.68	82.71	82.7	82.7	82.7	99.8	---	99.9	99.8	94.9	94.93	94.93	94.95	94.9	94.8	86	84.5
r34	82.72	82.7	82.76	82.71	82.75	82.7	82.7	82.8	99.8	99.9	---	99.8	94.9	94.93	94.93	94.95	94.9	94.7	86.02	84.6
ra97	82.78	82.8	82.81	82.77	82.76	82.7	82.8	82.8	99.8	99.8	99.8	---	94.9	94.92	94.95	94.96	95	94.7	86.12	84.6
r42	82.45	82.5	82.51	82.47	82.46	82.4	82.4	82.6	94.9	94.9	94.9	94.9	---	99.7	99.68	99.69	99.7	97.2	85.81	84.3
rrb103	82.4	82.4	82.45	82.41	82.44	82.4	82.4	82.6	94.9	94.9	94.9	94.9	99.7	---	99.77	99.76	99.9	97.2	85.8	84.4
rrb102	82.47	82.5	82.47	82.43	82.42	82.4	82.4	82.5	94.9	94.9	94.9	94.9	99.7	99.76	---	99.8	99.8	97.2	85.86	84.3
rrb32	82.48	82.5	82.48	82.44	82.42	82.4	82.4	82.6	94.9	94.9	94.9	94.9	99.7	99.76	99.8	---	99.8	97.2	85.87	84.4
rrb104	82.45	82.5	82.48	82.45	82.48	82.4	82.4	82.6	94.9	94.9	94.9	94.9	99.7	99.84	99.78	99.78	---	97.2	85.84	84.3
rrb33	82.43	82.4	82.46	82.42	82.44	82.4	82.4	82.5	94.7	94.8	94.7	94.8	97.2	97.22	97.19	97.2	97.2	---	85.79	84.2

Supplementary Table 2d. Pairwise AAI similarity scores from 255 core genes

	ch56	re10	re14	re11	re9	re12	re13	rd55	ra97	rrh31	r34	r38	rrb103	rrb32	rrb102	rrb104	r42	rrb33	rph87	rg82	r41	rrh29	rrh101	rp26	rp25	rp88	r111	r37	r93	rj52	r44	r43	ri20	ro86	ro24	rw47	ro85	rw96	ro23	ro22	rey98	r60	rey57	rey58	rey16	rq27	rq90	rq89	r105	rey99	rey17	rey19	rey15	rey59	rey18	ro84	rrh30	r107	r40	rrh92	r113	r108	rf61	rf69	rf70	rf64	rf65	r35	r36	rf75	r110	rf72	r114	rf63	rf73	rf74	rf81	rf62	rf66	rf76	rf100	rf67	rf68	rf77	rf78	rf79	rf80	r39	r112	r45	rc54	rkr83	r94	r109	r95	r106	rt46	rk21	rr28	rr91	na53	nb6	nc7	nf8	gb4	gp5	g3	cd2	cd1	w115	w116	sr48	srg49	smn50	tb51
ch56		99.3	99.37	99.42	99.37	99.4	99.6	93.6	85.3	85.2	85.3	85.3	85.3	85.28	85.25	85.31	85.3	85.3	85.01	83.9	83.81	84.23	83.9	83.7	83.8	84	84	83.7	83.98	86.6	86.6	86.5	86.1	86.3	86.19	86.6	86.6	86.5	86.5	86.4	85.6	85.61	85.66	85.39	85.6	85.7	85.7	85.4	85.6	85.7	85.7	85.78	85.65	85.64	85.66	85.57	85.41	85.6	85.5	85.5	85.3	85.4	82.8	82.9	82.9	82.9	82.8	82.9	82.9	82.4	82.1	82.5	82.4	82.3	82.4	82.4	82.4	82.3	82.3	82.3	82.4	82.3	82.3	82.3	82.3	82.4	82.4	82.4	82.4	82.2	81.25	81.4	81.3	81.2	86.5	85.6	85.8	81.92	82	70	80.6	80.8	80.8	80.7	75.3	75	76	65.6	65.2	75.9	76.3	68.2	68.3	76.95	76.5
re10	99.27		99.69	99.74	99.7	99.7	99.6	93.7	85.4	85.3	85.4	85.4	85.4	85.39	85.36	85.42	85.4	85.3	85.11	84.1	83.99	84.38	84.1	83.8	83.9	84.2	84.1	83.9	84.14	86.5	86.6	86.5	86.1	86.4	86.26	86.6	86.7	86.5	86.4	86.5	85.7	85.75	85.66	85.41	85.73	85.7	85.7	85.5	85.74	85.8	85.8	85.81	85.79	85.65	85.67	85.71	85.54	85.6	85.6	85.5	85.5	85.5	82.8	83	83	83	82.9	83	83	82.5	82.1	82.6	82.5	82.3	82.4	82.4	82.5	82.4	82.4	82.4	82.4	82.4	82.4	82.4	82.4	82.5	82.4	82.5	82.4	82.3	81.28	81.4	81.4	81.2	86.6	85.7	85.9	81.98	82	70	80.7	80.8	80.8	80.8	75.4	76	76	65.7	65.3	75.9	76.3	68.3	68.4	77.02	76.6
re14	99.37	99.7		99.82	99.83	99.8	99.7	93.7	85.4	85.4	85.4	85.4	85.4	85.46	85.43	85.49	85.5	85.3	85.15	84.1	83.98	84.4	84.1	83.8	83.9	84.2	84.1	83.9	84.12	86.6	86.7	86.5	86.2	86.5	86.33	86.7	86.7	86.6	86.5	86.5	85.7	85.78	85.7	85.44	85.77	85.74	85.7	85.5	85.77	85.8	85.8	85.85	85.83	85.68	85.71	85.75	85.58	85.6	85.6	85.5	85.5	85.5	82.8	83	83	83	82.9	83	82.9	82.5	82.1	82.5	82.5	82.3	82.5	82.4	82.5	82.4	82.4	82.4	82.5	82.4	82.4	82.4	82.4	82.4	82.4	82.5	82.4	82.2	81.34	81.5	81.4	81.3	86.6	85.7	85.9	82.03	82	70	80.6	80.8	80.8	80.8	75.3	76	76	65.7	65.3	75.9	76.3	68.3	68.4	77.01	76.7
re11	99.42	99.7	99.82		99.82	99.8	99.7	93.7	85.5	85.4	85.4	85.4	85.4	85.44	85.42	85.48	85.5	85.3	85.19	84.1	84.02	84.43	84.1	83.9	84	84.2	84.2	83.9	84.17	86.6	86.7	86.6	86.2	86.5	86.34	86.7	86.7	86.6	86.5	86.5	85.8	85.79	85.71	85.45	85.79	85.75	85.7	85.6	85.8	85.8	85.8	85.85	85.83	85.7	85.72	85.75	85.59	85.7	85.7	85.5	85.5	85.5	82.8	83	83	83	82.9	83	82.9	82.5	82.1	82.5	82.5	82.3	82.5	82.4	82.4	82.4	82.4	82.4	82.5	82.3	82.3	82.4	82.4	82.4	82.4	82.4	82.4	82.2	81.3	81.4	81.3	81.2	86.6	85.8	85.9	82.01	83	70	80.6	80.8	80.9	80.8	75.3	76	76	65.7	65.3	75.9	76.3	68.2	68.4	77.01	76.6
re9	99.37	99.7	99.83	99.82		100	99.7	93.7	85.5	85.4	85.4	85.5	85.5	85.48	85.46	85.51	85.5	85.3	85.18	84.1	84.02	84.43	84.1	83.9	84	84.2	84.2	83.9	84.16	86.6	86.7	86.5	86.2	86.5	86.32	86.7	86.8	86.6	86.5	86.6	85.8	85.83	85.74	85.48	85.81	85.78	85.8	85.6	85.82	85.8	85.9	85.88	85.87	85.73	85.75	85.79	85.62	85.7	85.7	85.6	85.6	85.5	82.8	83	83	83	82.9	83	83	82.5	82.1	82.6	82.5	82.4	82.4	82.5	82.5	82.4	82.4	82.4	82.5	82.4	82.4	82.4	82.4	82.5	82.4	82.5	82.4	82.3	81.3	81.4	81.4	81.2	86.7	85.8	86	82.01	83	70	80.7	80.8	80.9	80.8	75.4	76	76	65.7	65.3	75.9	76.4	68.4	68.3	77.06	76.6
re12	99.37	99.7	99.83	99.82	99.99		99.7	93.7	85.5	85.4	85.4	85.4	85.5	85.46	85.44	85.5	85.5	85.3	85.18	84.1	84.01	84.43	84.1	83.9	84	84.2	84.2	83.9	84.15	86.6	86.7	86.5	86.2	86.5	86.35	86.7	86.8	86.6	86.5	86.6	85.7	85.77	85.67	85.42	85.75	85.71	85.7	85.5	85.75	85.8	85.8	85.82	85.81	85.66	85.68	85.72	85.55	85.6	85.6	85.5	85.5	85.5	82.8	83	83	83	82.9	83	82.9	82.5	82.1	82.5	82.5	82.3	82.4	82.4	82.5	82.3	82.4	82.4	82.5	82.3	82.3	82.4	82.4	82.4	82.4	82.4	82.4	82.2	81.31	81.4	81.4	81.3	86.6	85.7	86	82.01	83	70	80.6	80.8	80.8	80.8	75.4	76	76	65.7	65.3	75.9	76.3	68.3	68.3	77.03	76.6
re13	99.6	99.6	99.65	99.69	99.66	99.7		93.9	85.5	85.4	85.4	85.4	85.4	85.44	85.42	85.48	85.5	85.4	85.15	84.1	83.98	84.38	84.1	83.8	83.9	84.2	84.1	83.9	84.13	86.7	86.7	86.6	86.2	86.5	86.35	86.8	86.7	86.6	86.6	86.6	85.7	85.77	85.8	85.55	85.75	85.86	85.8	85.5	85.77	85.9	85.8	85.96	85.82	85.8	85.82	85.75	85.58	85.8	85.7	85.6	85.5	85.6	82.9	83.1	83	83.1	83	83.1	83	82.5	82.2	82.6	82.6	82.4	82.5	82.5	82.5	82.4	82.4	82.4	82.5	82.4	82.4	82.4	82.4	82.5	82.5	82.5	82.5	82.3	81.41	81.5	81.4	81.3	86.7	85.8	86	82.07	83	70	80.7	80.9	80.9	80.8	75.4	76	76	65.7	65.3	76	76.4	68.3	68.4	77.09	76.7
rd55	93.62	93.7	93.72	93.73	93.74	93.7	93.9		85.2	85.1	85.2	85.2	85.1	85.11	85.11	85.16	85.1	85	84.84	83.8	83.67	83.92	84.1	83.5	84	83.8	83.8	83.5	83.75	86.7	86.7	86.7	86.3	86.5	86.38	86.8	86.8	86.6	86.6	86.5	85.8	85.86	85.92	85.67	85.87	85.99	85.9	85.6	85.89	86	85.9	86.12	85.92	85.95	85.96	85.85	85.64	85.9	85.8	85.5	85.5	85.6	82.8	82.8	82.8	82.9	82.8	82.8	82.8	82.7	82.4	82.8	82.7	82.6	82.7	82.7	82.7	82.7	82.7	82.6	82.8	82.6	82.6	82.7	82.6	82.7	82.8	82.8	82.7	82.5	81.26	81.4	81.4	81.4	86.8	85.8	85.8	81.64	82	70	80.5	80.5	80.8	80.6	75.1	75	75	65.4	65	75.9	76.1	68.2	68.6	77.24	76.6
ra97	85.28	85.4	85.44	85.45	85.48	85.5	85.4	85.2		99.8	99.9	99.9	97.3	97.42	97.26	97.4	97.3	96.9	89.66	88.3	88.11	88.63	88.3	88.1	88.3	88.4	88.4	88.1	88.43	85.4	85.5	85.4	85	85.2	85.07	85	85.5	85.4	85.2	85.2	84	84.05	83.98	83.71	84.02	84.04	84	83.3	84.04	84.1	84.1	84.14	84.09	84.02	84.02	83.98	83.78	84	83.9	83.6	83.6	83.6	81.6	81.7	81.7	81.7	81.6	81.7	81.6	81.5	80.6	81.6	81.6	81.3	81.4	81.5	81.5	81.4	81.3	81.4	81.4	81.3	81.3	81.4	81.4	81.4	81.4	81.5	81.4	81.2	80.82	81	80.8	80.6	84.4	83.9	84.2	81.41	82	70	80.1	80.3	80.5	80.5	75.1	75	75	65.9	65.5	75.7	76.2	68.1	68.5	76.89	76.2
rrh31	85.2	85.3	85.36	85.36	85.38	85.4	85.4	85.1	99.8		99.9	99.8	97.2	97.33	97.17	97.31	97.2	96.8	89.57	88.2	88.01	88.5	88.2	88	88.2	88.3	88.3	88	88.31	85.3	85.4	85.3	85	85.2	85	85.3	85.4	85.3	85.2	85.1	83.9	83.97	83.91	83.63	83.94	83.96	83.9	83.6	83.96	84	84	84.07	84.01	83.94	83.94	83.9	83.71	83.9	83.8	83.5	83.5	83.5	81.5	81.6	81.6	81.6	81.5	81.6	81.6	81.4	80.9	81.5	81.5	81.2	81.3	81.4	81.4	81.3	81.2	81.3	81.3	81.2	81.2	81.2	81.3	81.3	81.3	81.3	81.2	81.2	80.71	80.9	80.7	80.4	84.3	83.8	84.1	81.31	82	70	80	80.2	80.4	80.4	75.2	75	75	65.8	65.3	75.6	76.2	68.4	68.8	76.78	76.1
r34	85.25	85.4	85.41	85.41	85.44	85.4	85.4	85.2	99.9	99.9		99.9	97.2	97.4	97.24	97.38	97.3	96.9	89.63	88.3	88.07	88.58	88.2	88.1	88.2	88.4	88.4	88	88.37	85.3	85.4	85.3	85	85.2	85.03	85.3	85.4	85.3	85.2	85.2	84	84.01	83.94	83.67	83.98	84	83.9	83.7	84	84	84	84.1	84.05	83.98	83.98	83.94	83.74	83.9	83.8	83.6	83.6	83.6	81.6	81.7	81.7	81.6	81.6	81.7	81.6	81.5	81	81.6	81.6	81.3	81.4	81.4	81.5	81.3	81.3	81.4	81.4	81.3	81.3	81.3	81.4	81.4	81.4	81.4	81.3	81.2	80.79	80.9	80.7	80.5	84.4	83.9	84.2	81.35	82	70	80.1	80.3	80.4	80.5	75.3	75	76	65.9	65.4	75.7	76.2	68.4	68.8	76.85	76.2
r38	85.25	85.4	85.41	85.42	85.45	85.4	85.4	85.2	99.9	99.8	99.9		97.2	97.42	97.25	97.4	97.3	96.9	89.63	88.3	88.09	88.59	88.3	88.1	88.2	88.4	88.4	88	88.39	85.4	85.4	85.4	85	85.2	85.04	85.4	85.5	85.4	85.2	85.2	84	84.02	83.96	83.69	84	84.01	83.9	83.7	84.01	84	84	84.11	84.06	83.99	83.99	83.95	83.76	83.9	83.9	83.6	83.6	83.6	81.6	81.7	81.7	81.7	81.6	81.7	81.6	81.5	81	81.5	81.6	81.3	81.4	81.4	81.5	81.3	81.3	81.3	81.4	81.3	81.3	81.3	81.4	81.4	81.4	81.4	81.3	81.2	80.78	80.9	80.7	80.5	84.4	83.9	84.2	81.37	82	70	80.1	80.3	80.5	80.5	75.3	75	76	65.9	65.4	75.7	76.2	68.4	68.8	76.86	76.2
rrb103	85.26	85.4	85.44	85.42	85.46	85.5	85.4	85.1	97.3	97.2	97.2	97.2		99.7	99.84	99.74	99.5	98.1	89.61	88.1	87.98	88.46	88.1	87.9	88.1	88.2	88.2	87.9	88.18	85.1	85.2	85.1	84.8	85	84.86	85.1	85.3	85.2	85.1	85.1	83.8	83.85	83.79	83.51	83.84	83.87	83.9	83.5	83.88	83.9	83.9	83.99	83.9	83.83	83.84	83.81	83.6	83.8	83.8	83.5	83.5	83.5	81.5	81.6	81.6	81.6	81.5	81.6	81.6	81.4	81.2	81.5	81.5	81.2	81.4	81.4	81.4	81.3	81.3	81.3	81.3	81.3	81.2	81.3	81.3	81.3	81.3	81.4	81.3	81.1	80.84	80.9	80.6	80.5	84.2	83.8	84	81.22	82	70	79.9	80.2	80.4	80.4	75.3	75	75	65.8	65.4	75.6	76.1	68.3	68.7	76.68	76.1
rrb32	85.28	85.4	85.46	85.44	85.48	85.5	85.4	85.1	97.4	97.3	97.4	97.4	99.7		99.75	99.89	99.7	98.2	89.54	88.2	88.08	88.56	88.2	88	88.1	88.3	88.3	88	88.29	85.2	85.3	85.2	84.8	85	84.87	85.2	85.3	85.2	85.1	85.1	83.9	83.91	83.85	83.58	83.9	83.94	83.8	83.6	83.94	83.9	84	84.05	83.96	83.89	83.9	83.87	83.66	83.9	83.8	83.5	83.5	83.6	81.6	81.7	81.6	81.6	81.5	81.7	81.6	81.4	81.1	81.6	81.5	81.3	81.4	81.4	81.4	81.3	81.3	81.3	81.4	81.3	81.3	81.3	81.3	81.3	81.4	81.4	81.3	81.1	80.81	80.9	80.6	80.5	84.3	83.8	84	81.21	82	70	79.9	80.3	80.4	80.4	75.4	75	75	65.8	65.3	75.6	76.1	68.3	68.7	76.7	76.1
rrb102	85.25	85.4	85.43	85.42	85.46	85.4	85.4	85.1	97.3	97.2	97.2	97.3	99.8	99.75		99.72	99.6	98.1	89.62	88.1	87.98	88.47	88.1	87.9	88.1	88.2	88.2	87.9	88.17	85.1	85.3	85.1	84.8	85	84.86	85.1	85.3	85.2	85.1	85.1	83.8	83.86	83.79	83.52	83.84	83.87	83.9	83.6	83.88	83.9	83.9	83.98	83.9	83.83	83.83	83.8	83.6	83.8	83.7	83.5	83.5	83.5	81.6	81.7	81.6	81.7	81.5	81.7	81.6	81.4	81.1	81.5	81.5	81.2	81.3	81.4	81.4	81.3	81.3	81.3	81.3	81.3	81.3	81.3	81.3	81.3	81.3	81.4	81.3	81.1	80.81	80.9	80.6	80.5	84.2	83.8	84	81.21	82	70	79.9	80.2	80.4	80.4	75.4	75	75	65.8	65.4	75.6	76.1	68.3	68.7	76.68	76.1
rrb104	85.31	85.4	85.49	85.48	85.51	85.5	85.5	85.2	97.4	97.3	97.4	97.4	99.7	99.89	99.72		99.7	98.2	89.57	88.2	88.07	88.56	88.2	88	88.2	88.3	88.3	88	88.27	85.2	85.3	85.2	84.8	85.1	84.89	85.2	85.3	85.2	85.1	85.1	83.9	83.93	83.87	83.59	83.91	83.95	83.8	83.6	83.96	84	84	84.07	83.98	83.9	83.91	83.88	83.68	83.9	83.8	83.6	83.6	83.6	81.5	81.6	81.6	81.6	81.5	81.6	81.6	81.4	81.1	81.6	81.5	81.2	81.4	81.4	81.4	81.3	81.3	81.3	81.3	81.3	81.3	81.3	81.3	81.3	81.3	81.4	81.3	81.1	80.79	80.9	80.6	80.5	84.3	83.8	84.1	81.23	82	70	79.9	80.3	80.4	80.4	75.4	75	75	65.8	65.4	75.6	76.1	68.3	68.7	76.68	76.1
r42	[illegible]
rrb33	[illegible]
rph87	[illegible]
rg82	[illegible]
r41	[illegible]
rrh29	[illegible]
rrh101	[illegible]
rp26	[illegible]
rp25	[illegible]
rp88	[illegible]
r111	[illegible]
r37	[illegible]
r93	[illegible]
rj52	[illegible]
r44	[illegible]
r43	[illegible]
ri20	[illegible]
ro86	[illegible]
ro24	[illegible]
rw47	[illegible]
ro85	[illegible]
rw96	[illegible]
ro23	[illegible]
ro22	[illegible]
rey98	[illegible]
r60	[illegible]
rey57	[illegible]
rey58	[illegible]
rey16	[illegible]
rq27	[illegible]
rq90	[illegible]
rq89	[illegible]
r105	[illegible]
rey99	[illegible]
rey17	[illegible]
rey19	[illegible]
rey15	[illegible]
rey59	[illegible]
rey18	[illegible]
ro84	[illegible]
rrh30	[illegible]
r107	[illegible]
r40	[illegible]
rrh92	[illegible]
r113	[illegible]
r108	[illegible]
rf61	[illegible]
rf69	[illegible]
rf70	[illegible]
rf64	[illegible]
rf65	[illegible]
r35	[illegible]
r36	[illegible]
rf75	[illegible]
r110	[illegible]
rf72	[illegible]
r114	[illegible]
rf63	[illegible]
rf73	[illegible]
rf74	[illegible]
rf81	[illegible]
rf62	[illegible]
rf66	[illegible]
rf76	[illegible]
rf100	[illegible]
rf67	[illegible]
rf68	[illegible]
rf77	[illegible]
rf78	[illegible]
rf79	[illegible]
rf80	[illegible]
r39	[illegible]
r112	[illegible]
r45	[illegible]
rc54	[illegible]
rkr83	[illegible]
r94	[illegible]
r109	[illegible]
r95	[illegible]
r106	[illegible]
rt46	[illegible]
rk21	[illegible]
rr28	[illegible]
rr91	[illegible]
na53	[illegible]
nb6	[illegible]
nc7	[illegible]
nf8	[illegible]
gb4	[illegible]
gp5	[illegible]
g3	[illegible]
cd2	[illegible]
cd1	[illegible]
w115	[illegible]
w116	[illegible]
sr48	[illegible]
srg49	[illegible]
smn50	[illegible]
tb51	[illegible]

Supplementary Table 3. GGDC analyses within each phylogenetic group

Group A						
Query genome	Reference genome	DDH	Model C.I.	Bootstrap (	Distance	Prob. DDH >= 70%
NC_014659	LRRF00000000	93.1	1.71	93.1 - 93.1	0.0087	96.73
NC_014659	APJC00000000	91.5	1.40055	91.5 - 91.5	0.0105	96.26
NC_014659	LRQY00000000	93.1	1.71	93.1 - 93.1	0.0087	96.73
NC_014659	LRQZ00000000	90.8	2.02	90.8 - 90.8	0.0112	96.07
NC_014659	LRRA00000000	93.1	1.7	93.1 - 93.2	0.0087	96.74
NC_014659	NZ_CM001149	92	1.2636	92 - 92%	0.01	96.42
NC_014659	JPOC00000000	27	3.020253	27 - 27%	0.1603	0.03
Group B1-1						
Query genome	Reference genome	DDH	Model C.I.	Bootstrap (	Distance	Prob. DDH >= 70%
NZ_CP011341.1	LRRL00000000	44.6	2.56	44.6 - 44.6	0.0852	7.67
NZ_CP011341.1	LPZN00000000	21.4	2.34	21.4 - 21.4	0.2055	0
NZ_CP011341.1	LRRK00000000	21.6	2.35	21.6 - 21.7	0.2027	0
NZ_CP011341.1	LRRI00000000	21.5	2.34	21.5 - 21.5	0.2041	0
NZ_CP011341.1	AVAE00000000	92.2	2.575147	92.1 - 92.2	0.0098	96.47
NZ_CP011341.1	AZHI00000000	92.9	2.791065	92.9 - 92.9	0.0089	96.68
NZ_CP011341.1	CAVJ000000000	94.5	2.854621	94.5 - 94.5	0.0072	97.08
NZ_CP011341.1	AJVB00000000	22	2.920821	22 - 22%	0.1994	0
NZ_CP011341.1	AGVW00000000	21.6	2.966245	21.6 - 21.6	0.2032	0
NZ_CP011341.1	AFAQ00000000	21.4	2.888954	21.4 - 21.4	0.2051	0
NZ_CP011341.1	AHBW00000000	21.6	2.966435	21.6 - 21.6	0.2031	0
NZ_CP011341.1	JPJJ01000000	21.5	2.961796	21.5 - 21.5	0.2045	0
NZ_CP011341.1	JTIZ01000000	21.5	2.957577	21.5 - 21.5	0.2045	0
NZ_CP011341.1	NC_023150	21.5	2.97309	21.5 - 21.5	0.2042	0
NZ_CP011341.1	LRRH00000000	22.7	2.37	22.7 - 22.7	0.1932	0
NZ_CP011341.1	AJFC00000000	45	3.333403	45 - 45%	0.0844	8.16
NZ_CP011341.1	CCSD01000000	44.8	3.306344	44.7 - 44.8	0.0849	7.83
NZ_CP011341.1	LDUF01000000	44.8	3.327868	44.8 - 44.8	0.0848	7.89
NZ_CP011341.1	ANGC00000000	44.7	3.339426	44.7 - 44.7	0.0852	7.68
NZ_CP011341.1	AOEX00000000	43.5	3.256966	43.5 - 43.5	0.0886	6.05
Group B1-2						
Query genome	Reference genome	DDH	Model C.I.	Bootstrap (	Distance	Prob. DDH >= 70%
LRRL00000000	AVAE00000000	44.1	2.55	44.1 - 44.1	0.0869	6.83
LRRL00000000	AFAQ00000000	21.5	2.34	21.5 - 21.5	0.2046	0
LRRL00000000	CAVJ000000000	44.9	2.56	44.9 - 44.9	0.0844	8.11
LRRL00000000	AJVB00000000	22	2.35	22 - 22%	0.1993	0
LRRL00000000	AJFC00000000	93	1.73	93 - 93%	0.0089	96.69
LRRL00000000	JPJJ01000000	21.6	2.35	21.6 - 21.6	0.2028	0
LRRL00000000	JTIZ01000000	21.6	2.35	21.6 - 21.6	0.203	0
LRRL00000000	NZ_CP011341.1	44.6	2.56	44.6 - 44.6	0.0852	7.67
LRRL00000000	LPZN00000000	21.4	2.34	21.4 - 21.4	0.2056	0
LRRL00000000	NC_023150	21.6	2.35	21.6 - 21.6	0.2028	0
LRRL00000000	AHBW00000000	21.8	2.35	21.8 - 21.8	0.2015	0
LRRL00000000	LRRI00000000	21.5	2.34	21.5 - 21.5	0.2037	0
LRRL00000000	LRRH00000000	22.6	2.36	22.6 - 22.6	0.1936	0

LRRL00000000	ANGC00000000	94.8	1.43	94.8 - 94.8	0.0068	97.16
LRRL00000000	AOEX00000000	60.3	2.82	60.3 - 60.3	0.0511	53.34
LRRL00000000	CCSD01000000	92.2	1.84	92.2 - 92.2	0.0097	96.48
LRRL00000000	LDUF01000000	92.9	1.74	92.9 - 92.9	0.009	96.68
LRRL00000000	AGVW00000000	21.7	2.35	21.7 - 21.7	0.2026	0
LRRL00000000	AZHI00000000	44.1	2.55	44.1 - 44.1	0.0869	6.84
LRRL00000000	LRRK00000000	21.7	2.35	21.7 - 21.7	0.2026	0
Group B1-3						
Query genome	Reference genome	DDH	Model C.I.	Bootstrap (	Distance	Prob. DDH >= 70%
AOEX00000000	AVAE00000000	42.7	[40.1 - 45.2	42.7 - 42.7	0.091	5.07
AOEX00000000	AFAQ00000000	21.3	[19.1 - 23.7	21.3 - 21.3	0.2059	0
AOEX00000000	CAVJ000000000	43.3	[40.8 - 45.9	43.3 - 43.3	0.089	5.84
AOEX00000000	AJVB00000000	21.8	[19.6 - 24.3	21.8 - 21.9	0.2008	0
AOEX00000000	AJFC00000000	60.6	[57.8 - 63.4	60.6 - 60.6	0.0505	54.46
AOEX00000000	JPJJ01000000	21.6	[19.3 - 24%	21.6 - 21.6	0.2035	0
AOEX00000000	JTIZ01000000	21.4	[19.1 - 23.8	21.4 - 21.4	0.2054	0
AOEX00000000	NZ_CP011341.1	43.5	[41 - 46%]	43.5 - 43.5	0.0886	6.05
AOEX00000000	LPZN00000000	21.4	[19.1 - 23.8	21.4 - 21.4	0.2052	0
AOEX00000000	NC_023150	21.2	[19 - 23.6%	21.2 - 21.2	0.2071	0
AOEX00000000	AHBW00000000	21.4	[19.1 - 23.8	21.4 - 21.4	0.2052	0
AOEX00000000	LRRI00000000	21.4	[19.1 - 23.8	21.4 - 21.4	0.2052	0
AOEX00000000	LRRH00000000	22.2	[20 - 24.7%	22.2 - 22.2	0.1973	0
AOEX00000000	ANGC00000000	59.6	[56.8 - 62.4	59.6 - 59.6	0.0523	51.08
AOEX00000000	LRRL00000000	60.3	[57.5 - 63.1	60.3 - 60.3	0.0511	53.34
AOEX00000000	CCSD01000000	60	[57.2 - 62.8	60 - 60%	0.0516	52.39
AOEX00000000	LDUF01000000	60.5	[57.6 - 63.2	60.5 - 60.5	0.0508	53.87
AOEX00000000	AGVW00000000	21.5	[19.2 - 23.9	21.4 - 21.5	0.2046	0
AOEX00000000	AZHI00000000	42.9	[40.4 - 45.5	42.9 - 42.9	0.0902	5.37
AOEX00000000	LRRK00000000	21.4	[19.2 - 23.9	21.4 - 21.4	0.2049	0
Group B-4						
Query genome	Reference genome	DDH	Model C.I.	Bootstrap (	Distance	Prob. DDH >= 70%
LRRH00000000	AVAE00000000	22.9	2.37	22.9 - 22.9	0.1915	0
LRRH00000000	AFAQ00000000	22	2.35	22 - 22%	0.1996	0
LRRH00000000	CAVJ000000000	22.9	2.37	22.9 - 22.9	0.1915	0
LRRH00000000	AJVB00000000	22.6	2.36	22.5 - 22.6	0.1943	0
LRRH00000000	AJFC00000000	22.7	2.37	22.7 - 22.7	0.1928	0
LRRH00000000	JPJJ01000000	22.2	2.36	22.2 - 22.2	0.1978	0
LRRH00000000	JTIZ01000000	22.2	2.36	22.2 - 22.2	0.1976	0
LRRH00000000	NZ_CP011341.1	22.7	2.37	22.7 - 22.7	0.1932	0
LRRH00000000	LPZN00000000	22	2.35	22 - 22%	0.1993	0
LRRH00000000	NC_023150	21.9	2.35	21.9 - 21.9	0.2007	0
LRRH00000000	AHBW00000000	22	2.35	22 - 22%	0.1995	0
LRRH00000000	LRRI00000000	21.9	2.35	21.9 - 21.9	0.1999	0
LRRH00000000	ANGC00000000	22.7	2.37	22.7 - 22.7	0.1933	0
LRRH00000000	AOEX00000000	22.2	2.36	22.2 - 22.2	0.1973	0
LRRH00000000	LRRL00000000	22.6	2.36	22.6 - 22.6	0.1936	0
LRRH00000000	CCSD01000000	22.6	2.37	22.6 - 22.7	0.1934	0
LRRH00000000	LDUF01000000	22.6	2.36	22.6 - 22.6	0.1939	0

LRRH00000000	AGVW00000000	22.4	2.36	22.4 - 22.4	0.1959	0
LRRH00000000	AZHI00000000	23	2.37	23 - 23%	0.1903	0
LRRH00000000	LRRK00000000	22	2.35	22 - 22%	0.1993	0
Group B2-5						
Query genome	Reference genome	DDH	Model C.I.	Bootstrap (	Distance	Prob. DDH >= 70%
LPZN00000000	AVAE00000000	21.8	2.35	21.8 - 21.8	0.201	0
LPZN00000000	AFAQ00000000	41.9	2.53	41.9 - 41.9	0.0932	4.3
LPZN00000000	CAVJ000000000	21.8	2.35	21.8 - 21.8	0.2008	0
LPZN00000000	AJVB00000000	85.7	2.49	85.7 - 85.7	0.0168	94.07
LPZN00000000	AJFC00000000	21.6	2.34	21.6 - 21.6	0.2033	0
LPZN00000000	JPJJ01000000	42.5	2.53	42.5 - 42.5	0.0914	4.91
LPZN00000000	JTIZ01000000	42	2.53	41.9 - 42%	0.0931	4.33
LPZN00000000	NZ_CP011341.1	21.4	2.34	21.4 - 21.4	0.2055	0
LPZN00000000	NC_023150	42.2	2.53	42.2 - 42.2	0.0923	4.62
LPZN00000000	AHBW00000000	43	2.54	43 - 43%	0.09	5.44
LPZN00000000	LRRI00000000	43	2.54	43 - 43%	0.0899	5.5
LPZN00000000	LRRH00000000	22	2.35	22 - 22%	0.1993	0
LPZN00000000	ANGC00000000	21.6	2.34	21.6 - 21.6	0.2033	0
LPZN00000000	AOEX00000000	21.4	2.34	21.4 - 21.4	0.2052	0
LPZN00000000	LRRL00000000	21.4	2.34	21.4 - 21.4	0.2056	0
LPZN00000000	CCSD01000000	21.6	2.34	21.6 - 21.6	0.2035	0
LPZN00000000	LDUF01000000	21.7	2.35	21.7 - 21.7	0.2019	0
LPZN00000000	AGVW00000000	41.8	2.52	41.8 - 41.8	0.0936	4.19
LPZN00000000	AZHI00000000	21.8	2.35	21.8 - 21.8	0.2016	0
LPZN00000000	LRRK00000000	41.7	2.52	41.7 - 41.7	0.0938	4.12
Group B2-6a						
Query genome	Reference genome	DDH	Model C.I.	Bootstrap (	Distance	Prob. DDH >= 70%
LRRK00000000	AVAE00000000	21.8	2.35	21.8 - 21.8	0.2013	0
LRRK00000000	AFAQ00000000	57.1	2.76	57.1 - 57.1	0.0568	42.4
LRRK00000000	CAVJ000000000	21.8	2.35	21.8 - 21.8	0.2009	0
LRRK00000000	AJVB00000000	41.6	2.52	41.6 - 41.6	0.0944	3.95
LRRK00000000	AJFC00000000	21.8	2.35	21.8 - 21.8	0.2009	0
LRRK00000000	JPJJ01000000	59.3	2.8	59.3 - 59.3	0.0528	50.04
LRRK00000000	JTIZ01000000	59.1	2.79	59.1 - 59.1	0.0532	49.24
LRRK00000000	NZ_CP011341.1	21.6	2.35	21.6 - 21.7	0.2027	0
LRRK00000000	LPZN00000000	41.7	2.52	41.7 - 41.7	0.0938	4.12
LRRK00000000	NC_023150	59.2	2.8	59.2 - 59.2	0.0531	49.54
LRRK00000000	AHBW00000000	58.9	2.79	58.9 - 58.9	0.0535	48.76
LRRK00000000	LRRI00000000	58.7	2.79	58.7 - 58.7	0.0539	47.89
LRRK00000000	LRRH00000000	22	2.35	22 - 22%	0.1993	0
LRRK00000000	ANGC00000000	21.6	2.35	21.6 - 21.6	0.2027	0
LRRK00000000	AOEX00000000	21.4	2.34	21.4 - 21.4	0.2049	0
LRRK00000000	LRRL00000000	21.7	2.35	21.7 - 21.7	0.2026	0
LRRK00000000	CCSD01000000	21.6	2.35	21.6 - 21.6	0.2029	0
LRRK00000000	LDUF01000000	21.5	2.34	21.5 - 21.5	0.2043	0
LRRK00000000	AGVW00000000	62.5	2.85	62.5 - 62.6	0.0473	60.44
LRRK00000000	AZHI00000000	21.8	2.35	21.8 - 21.8	0.2013	0

Group B2-6b						
Query genome	Reference genome	DDH	Model C.I.	Bootstrap (	Distance	Prob. DDH >= 70%
AGVW00000000	LPZN00000000	41.8	2.52	41.8 - 41.8	0.0936	4.19
AGVW00000000	LRRI00000000	59.9	2.81	59.9 - 59.9	0.0517	52.1
AGVW00000000	LRRH00000000	22.4	2.36	22.4 - 22.4	0.1959	0
AGVW00000000	LRRL00000000	21.7	2.35	21.7 - 21.7	0.2026	0
AGVW00000000	LRRK00000000	62.5	2.85	62.5 - 62.6	0.0473	60.44
AGVW00000000	AVAE00000000	21.7	2.950495	21.7 - 21.7	0.2018	0
AGVW00000000	CAVJ000000000	21.7	2.942169	21.7 - 21.7	0.2018	0
AGVW00000000	NZ_CP011341.1	21.6	2.966245	21.6 - 21.6	0.2032	0
AGVW00000000	AZHI00000000	21.7	2.943735	21.7 - 21.7	0.202	0
AGVW00000000	AJFC00000000	21.8	2.935339	21.8 - 21.8	0.2013	0
AGVW00000000	CCSD01000000	21.7	2.942023	21.7 - 21.7	0.2021	0
AGVW00000000	LDUF01000000	21.7	2.951113	21.7 - 21.7	0.2026	0
AGVW00000000	ANGC00000000	21.7	2.949395	21.7 - 21.7	0.2021	0
AGVW00000000	AOEX00000000	21.5	2.942441	21.4 - 21.5	0.2046	0
AGVW00000000	AJVB00000000	42	3.102589	42 - 42%	0.0931	4.34
AGVW00000000	JTIZ01000000	59.3	3.199612	59.3 - 59.3	0.0529	49.9
AGVW00000000	AFAQ00000000	58.7	3.005453	58.7 - 58.7	0.0539	47.91
AGVW00000000	JPJJ01000000	61.2	3.219722	61.2 - 61.2	0.0495	56.38
AGVW00000000	NC_023150	60.8	3.294971	60.8 - 60.8	0.0503	54.86
AGVW00000000	AHBW00000000	60.2	3.224831	60.2 - 60.2	0.0513	52.88
Group B2-7						
Query genome	Reference genome	DDH	Model C.I.	Bootstrap (	Distance	Prob. DDH >= 70%
LRRI00000000	AVAE00000000	22	2.35	22 - 22%	0.199	0
LRRI00000000	AFAQ00000000	84.2	2.59	84.1 - 84.2	0.0186	93.29
LRRI00000000	CAVJ000000000	22.1	2.35	22.1 - 22.1	0.1983	0
LRRI00000000	AJVB00000000	42.8	2.54	42.8 - 42.8	0.0905	5.25
LRRI00000000	AJFC00000000	21.8	2.35	21.8 - 21.8	0.2017	0
LRRI00000000	JPJJ01000000	86.9	2.41	86.8 - 86.9	0.0156	94.59
LRRI00000000	JTIZ01000000	87.4	2.36	87.4 - 87.4	0.015	94.82
LRRI00000000	NZ_CP011341.1	21.5	2.34	21.5 - 21.5	0.2041	0
LRRI00000000	LPZN00000000	43	2.54	43 - 43%	0.0899	5.5
LRRI00000000	NC_023150	86.4	2.44	86.4 - 86.4	0.0161	94.38
LRRI00000000	AHBW00000000	87.8	2.32	87.8 - 87.8	0.0145	94.99
LRRI00000000	LRRH00000000	21.9	2.35	21.9 - 21.9	0.1999	0
LRRI00000000	ANGC00000000	21.7	2.35	21.7 - 21.7	0.2025	0
LRRI00000000	AOEX00000000	21.4	2.34	21.4 - 21.4	0.2052	0
LRRI00000000	LRRL00000000	21.5	2.34	21.5 - 21.5	0.2037	0
LRRI00000000	CCSD01000000	21.7	2.35	21.7 - 21.7	0.2018	0
LRRI00000000	LDUF01000000	21.8	2.35	21.8 - 21.8	0.2016	0
LRRI00000000	AGVW00000000	59.9	2.81	59.9 - 59.9	0.0517	52.1
LRRI00000000	AZHI00000000	22	2.35	22 - 22%	0.1992	0
LRRI00000000	LRRK00000000	58.7	2.79	58.7 - 58.7	0.0539	47.89
Group C-1						
Query genome	Reference genome	DDH	Model C.I.	Bootstrap (	Distance	Prob. DDH >= 70%
NC_008268	LRRG00000000	60.3	2.82	60.3 - 60.3	0.0511	53.41
NC_008268	AKKP00000000	90.3	3.284781	90.3 - 90.3	0.0118	95.89

NC_008268	AJLQ00000000	93.9	2.847251	93.9 - 94%	0.0078	96.95
NC_008268	NC_008268	100	Inf	100 - 100%	0	98.3
NC_008268	AJJH00000000	60.4	3.143948	60.4 - 60.4	0.0509	53.65
NC_008268	AJYC00000000	59.8	3.227206	59.8 - 59.8	0.0519	51.78
NC_008268	ANIU00000000	60.1	3.16812	60.1 - 60.1	0.0514	52.82
NC_008268	CP008947.1	58.7	3.333428	58.7 - 58.7	0.0538	48.09
NC_008268	BAWF01000000	58.5	3.135921	58.5 - 58.5	0.0543	47.12
NC_008268	AGVD00000000	60	3.280012	60 - 60%	0.0515	52.48
NC_008268	NC_012522	42	3.123612	42 - 42%	0.0929	4.42
Group C-2						
Query genome	Reference genome	DDH	Model C.I.	Bootstrap (	Distance	Prob. DDH >= 70%
LRRG00000000	AKKP00000000	60.1	2.81	60.1 - 60.1	0.0515	52.57
LRRG00000000	AJLQ00000000	59.5	2.8	59.4 - 59.5	0.0526	50.5
LRRG00000000	AJJH00000000	81.2	2.74	81.2 - 81.2	0.0219	91.47
LRRG00000000	NC_008268	60.3	2.82	60.3 - 60.3	0.0511	53.41
LRRG00000000	NC_012522	42.8	2.53	42.8 - 42.8	0.0907	5.18
LRRG00000000	AGVD00000000	70.6	2.93	70.6 - 70.6	0.0352	79.46
LRRG00000000	AJYC00000000	85.2	2.53	85.2 - 85.2	0.0174	93.83
LRRG00000000	CP008947.1	59.2	2.8	59.2 - 59.2	0.053	49.6
LRRG00000000	ANIU00000000	97.1	0.96	97.1 - 97.1	0.0042	97.67
LRRG00000000	BAWF01000000	58.2	2.78	58.2 - 58.2	0.0549	46.13
Group C-3						
Query genome	Reference genome	DDH	Model C.I.	Bootstrap (	Distance	Prob. DDH >= 70%
BAWF01000000	AKKP00000000	58.2	2.78	58.2 - 58.2	0.0548	46.17
BAWF01000000	AJLQ00000000	57.1	2.76	57.1 - 57.1	0.0569	42.26
BAWF01000000	AJJH00000000	57.4	2.76	57.4 - 57.4	0.0563	43.39
BAWF01000000	NC_008268	58.5	2.78	58.5 - 58.5	0.0543	47.12
BAWF01000000	NC_012522	43.5	2.54	43.5 - 43.5	0.0884	6.12
BAWF01000000	AGVD00000000	58.5	2.78	58.5 - 58.6	0.0542	47.41
BAWF01000000	AJYC00000000	57.8	2.77	57.8 - 57.8	0.0555	44.83
BAWF01000000	CP008947.1	91.6	1.92	91.6 - 91.6	0.0104	96.31
BAWF01000000	LRRG00000000	58.2	2.78	58.2 - 58.2	0.0549	46.13
BAWF01000000	ANIU00000000	57.5	2.76	57.5 - 57.5	0.0562	43.65
Group C-4						
Query genome	Reference genome	DDH	Model C.I.	Bootstrap (	Distance	Prob. DDH >= 70%
AGVD00000000	AKKP00000000	59.8	2.81	59.8 - 59.8	0.052	51.64
AGVD00000000	AJLQ00000000	59.2	2.8	59.2 - 59.2	0.053	49.77
AGVD00000000	AJJH00000000	69.2	2.92	69.2 - 69.2	0.0373	76.75
AGVD00000000	NC_008268	60	2.81	60 - 60%	0.0515	52.48
AGVD00000000	NC_012522	43.2	2.54	43.2 - 43.2	0.0894	5.71
AGVD00000000	AJYC00000000	69.8	2.92	69.8 - 69.8	0.0363	78.03
AGVD00000000	CP008947.1	59.1	2.8	59.1 - 59.2	0.0531	49.47
AGVD00000000	LRRG00000000	70.6	2.93	70.6 - 70.6	0.0352	79.46
AGVD00000000	ANIU00000000	70	2.92	70 - 70%	0.0362	78.27
AGVD00000000	BAWF01000000	58.5	2.78	58.5 - 58.6	0.0542	47.41
Group C-5						

Query genome	Reference genome	DDH	Model C.I.	Bootstrap (	Distance	Prob. DDH >= 70%
NC_012522	LRRG00000000	42.8	2.53	42.8 - 42.8	0.0907	5.18
NC_012522	AKKP00000000	42.4	3.066658	42.4 - 42.4	0.0917	4.81
NC_012522	AJLQ00000000	42.1	3.056392	42.1 - 42.1	0.0928	4.46
NC_012522	NC_008268	42	3.123612	42 - 42%	0.0929	4.42
NC_012522	AJJH00000000	42.8	3.017644	42.8 - 42.8	0.0906	5.23
NC_012522	AJYC00000000	42.6	3.039651	42.6 - 42.6	0.0911	5.04
NC_012522	ANIU00000000	43.2	3.033316	43.2 - 43.2	0.0894	5.7
NC_012522	CP008947.1	43.6	3.143554	43.6 - 43.6	0.0882	6.19
NC_012522	BAWF01000000	43.5	3.035224	43.5 - 43.5	0.0884	6.12
NC_012522	AGVD00000000	43.2	3.079909	43.2 - 43.2	0.0894	5.71
Group D-1						
Query genome	Reference genome	DDH	Model C.I.	Bootstrap (	Distance	Prob. DDH >= 70%
LRRJ00000000	AVCO00000000	63.2	[60.3 - 66%	63.2 - 63.2	0.0463	62.34
LRRJ00000000	CFHW01000000	83.9	[81.1 - 86.3	83.9 - 83.9	0.0189	93.14
LRRJ00000000	LEKE01000000	62.6	[59.7 - 65.4	62.6 - 62.6	0.0472	60.62
LRRJ00000000	NC_012490	62.8	[59.9 - 65.6	62.8 - 62.8	0.047	61.11
LRRJ00000000	ACNO00000000	84.8	[82 - 87.1%	84.7 - 84.8	0.0179	93.6
LRRJ00000000	NC_022115	62.9	[60 - 65.7%	62.9 - 62.9	0.0468	61.38
LRRJ00000000	AGCF00000000	62.4	[59.5 - 65.2	62.4 - 62.4	0.0475	60.02
LRRJ00000000	AUZK00000000	62.7	[59.8 - 65.5	62.7 - 62.7	0.0471	60.8
LRRJ00000000	BBLL01000000	88.5	[86 - 90.6%	88.5 - 88.5	0.0138	95.25
LRRJ00000000	JOIL01000000	80	[77.1 - 82.7	80 - 80%	0.0233	90.6
LRRJ00000000	ASKF01000000	62	[59.1 - 64.7	61.9 - 62%	0.0483	58.62
LRRJ00000000	LPZM00000000	99.9	[99.9 - 100	99.9 - 99.9	0.0002	98.28
LRRJ00000000	LDPN01000000	87.3	[84.7 - 89.5	87.3 - 87.3	0.0151	94.77
LRRJ00000000	NZ_CP011295.1	62.7	[59.8 - 65.5	62.7 - 62.7	0.047	60.97
LRRJ00000000	JOIM01000000	62.9	[60 - 65.7%	62.9 - 62.9	0.0467	61.56
LRRJ00000000	AODN00000000	88.8	[86.4 - 90.8	88.8 - 88.8	0.0134	95.37
LRRJ00000000	JNCU01000000	87	[84.4 - 89.2	87 - 87%	0.0154	94.64
LRRJ00000000	ASJJ00000000	62.8	[59.9 - 65.7	62.8 - 62.8	0.0468	61.32
LRRJ00000000	JYOP01000000	24.1	[21.8 - 26.5	24.1 - 24.1	0.1813	0.01
LRRJ00000000	CEDU01000000	24.2	[21.9 - 26.6	24.2 - 24.2	0.1807	0.01
LRRJ00000000	JNWS01000000	24.2	[21.9 - 26.7	24.2 - 24.2	0.18	0.01
Group D-2						
Query genome	Reference genome	DDH	Model C.I.	Bootstrap (	Distance	Prob. DDH >= 70%
NC_022115	LPZM00000000	62.9	2.86	62.9 - 62.9	0.0468	61.44
NC_022115	LRRJ00000000	62.9	2.86	62.9 - 62.9	0.0468	61.38
NC_022115	CFHW01000000	61.8	2.745462	61.8 - 61.8	0.0486	58.09
NC_022115	ACNO00000000	63	2.989471	63 - 63%	0.0466	61.74
NC_022115	BBLL01000000	62.9	3.185965	62.9 - 62.9	0.0467	61.59
NC_022115	JOIL01000000	68.9	2.889865	68.9 - 68.9	0.0377	76.19
NC_022115	LDPN01000000	62.8	3.253618	62.8 - 62.8	0.0469	61.21
NC_022115	AODN00000000	63.2	2.893775	63.2 - 63.2	0.0463	62.26
NC_022115	JNCU01000000	62.6	3.3085	62.6 - 62.6	0.0472	60.7
NC_022115	LEKE01000000	88.2	1.808672	88.2 - 88.2	0.0141	95.13
NC_022115	AVCO00000000	88.8	2.582524	88.8 - 88.8	0.0135	95.37
NC_022115	NC_012490	88.3	1.901906	88.3 - 88.3	0.014	95.19

NC_022115	AGCF00000000	88.3	2.889517	88.3 - 88.3	0.014	95.18
NC_022115	AUZK00000000	87.6	2.25441	87.6 - 87.6	0.0147	94.92
NC_022115	ASKF01000000	89.1	2.133519	89.1 - 89.1	0.0131	95.48
NC_022115	NZ_CP011295.1	89	1.339991	89 - 89%	0.0132	95.45
NC_022115	JOIM01000000	100	0.089949	99.9 - 99.9	0.0003	98.26
NC_022115	ASJJ00000000	100	1.05517	99.6 - 99.6	0.0008	98.19
NC_022115	JYOP01000000	24.1	[21.8 - 26.5	24.1 - 24.1	0.1815	0.01
NC_022115	CEDU01000000	24	[21.7 - 26.5	24 - 24%	0.1819	0
NC_022115	JNWS01000000	24.1	[21.8 - 26.5	24.1 - 24.1	0.1814	0.01
Group D-3						
Query genome	Reference genome	DDH	Model C.I.	Bootstrap (	Distance	Prob. DDH >= 70%
JNWS01000000	JYOP01000000	88.8	2.862449	88.8 - 88.8	0.0134	95.37
JNWS01000000	CEDU01000000	83.4	2.723753	83.4 - 83.4	0.0194	92.85
Group E1-1						
Query genome	Reference genome	DDH	Model C.I.	Bootstrap (	Distance	Prob. DDH >= 70%
JOKB01000000	ARTN00000000	56.8	3.348659	56.8 - 56.8	0.0575	41.15
JOKB01000000	ARND00000000	-9999			-9999	
JOKB01000000	JMEX01000000	57	3.327823	57 - 57.1%	0.0569	42.17
JOKB01000000	JMEW01000000	59.3	3.348787	59.3 - 59.3	0.0528	50.12
JOKB01000000	JMFF01000000	57.3	3.348982	57.3 - 57.3	0.0565	43.01
JOKB01000000	JMFE01000000	57.5	3.332635	57.5 - 57.5	0.0562	43.62
JOKB01000000	JMEV01000000	22	2.899647	22 - 22%	0.1992	0
JOKB01000000	BAOX01000000	21.3	2.821507	21.3 - 21.3	0.2064	0
JOKB01000000	NZ_CP010797.1	20.2	2.875993	20.2 - 20.2	0.2175	0
JOKB01000000	JMFC01000000	22.5	2.907818	22.5 - 22.5	0.1946	0
JOKB01000000	JMFD01000000	20.6	2.840616	20.6 - 20.6	0.2134	0
JOKB01000000	JMFA01000000	21.3	2.868488	21.3 - 21.3	0.2062	0
JOKB01000000	JMEZ01000000	20.9	2.863237	20.9 - 20.9	0.2103	0
JOKB01000000	JMEM01000000	22.1	2.878388	22.1 - 22.1	0.1987	0
JOKB01000000	JZIS01000000	20.6	2.865046	20.6 - 20.6	0.213	0
JOKB01000000	AXVF00000000	20.6	2.878426	20.6 - 20.6	0.2132	0
JOKB01000000	LFDS01000000	20.4	2.869638	20.4 - 20.4	0.2153	0
JOKB01000000	JMEY01000000	23	2.894318	23 - 23%	0.1906	0
JOKB01000000	JMEU01000000	22.9	2.910638	22.9 - 22.9	0.1915	0
JOKB01000000	JMES01000000	20.5	2.859485	20.5 - 20.5	0.2144	0
JOKB01000000	JMER01000000	20.6	2.857774	20.6 - 20.6	0.2129	0
JOKB01000000	JMET01000000	22.5	2.901807	22.5 - 22.5	0.195	0
JOKB01000000	JMEQ01000000	20.7	2.865869	20.7 - 20.7	0.2124	0
JOKB01000000	JMEP01000000	22.5	2.901084	22.5 - 22.5	0.1945	0
JOKB01000000	JMEO01000000	23.1	2.899508	23.1 - 23.1	0.1894	0
JOKB01000000	JMEN01000000	22.6	2.898779	22.6 - 22.6	0.1942	0
JOKB01000000	CAPS00000000	21.6	2.830852	21.6 - 21.6	0.2032	0
Group E1-2						
Query genome	Reference genome	DDH	Model C.I.	Bootstrap (	Distance	Prob. DDH >= 70%
ARTN00000000	JOKB01000000	56.8	3.348659	56.8 - 56.8	0.0575	41.15
ARTN00000000	ARND00000000	-9999			-9999	
ARTN00000000	JMEX01000000	91.6	2.838378	91.6 - 91.6	0.0103	96.31

ARTN00000000	JMEW01000000	88.1	3.239821	88 - 88.1%	0.0142	95.09
ARTN00000000	JMFF01000000	92.9	2.948312	92.9 - 92.9	0.009	96.67
ARTN00000000	JMFE01000000	91.9	3.036118	91.9 - 91.9	0.01	96.4
ARTN00000000	JMEV01000000	20.2	2.888275	20.2 - 20.2	0.2178	0
ARTN00000000	BAOX01000000	20.9	2.826377	20.9 - 21%	0.2097	0
ARTN00000000	NZ_CP010797.1	20.1	2.903683	20.1 - 20.1	0.2183	0
ARTN00000000	JMFC01000000	20.1	2.887823	20.1 - 20.1	0.2191	0
ARTN00000000	JMFD01000000	20.4	2.854134	20.4 - 20.4	0.2157	0
ARTN00000000	JMFA01000000	20.3	2.857897	20.3 - 20.3	0.2161	0
ARTN00000000	JMEZ01000000	20.2	2.862233	20.2 - 20.2	0.2172	0
ARTN00000000	JMEM01000000	20.3	2.856197	20.3 - 20.3	0.2169	0
ARTN00000000	JZIS01000000	20.3	2.885933	20.3 - 20.3	0.2163	0
ARTN00000000	AXVF00000000	20.2	2.890538	20.2 - 20.2	0.2176	0
ARTN00000000	LFDS01000000	20.2	2.886474	20.2 - 20.2	0.2179	0
ARTN00000000	JMEY01000000	20.5	2.860448	20.5 - 20.5	0.2144	0
ARTN00000000	JMEU01000000	20.2	2.875665	20.2 - 20.2	0.2173	0
ARTN00000000	JMES01000000	20.3	2.86866	20.3 - 20.3	0.217	0
ARTN00000000	JMER01000000	20.3	2.871721	20.3 - 20.3	0.2169	0
ARTN00000000	JMET01000000	20.2	2.875074	20.2 - 20.2	0.2174	0
ARTN00000000	JMEQ01000000	20.3	2.870311	20.3 - 20.3	0.2164	0
ARTN00000000	JMEP01000000	20.2	2.873651	20.2 - 20.2	0.2175	0
ARTN00000000	JMEO01000000	20.3	2.866434	20.3 - 20.3	0.2168	0
ARTN00000000	JMEN01000000	20.3	2.869777	20.3 - 20.4	0.216	0
ARTN00000000	CAPS00000000	20.6	2.828702	20.6 - 20.6	0.2136	0
Group E2-3						
Query genome	Reference genome	DDH	Model C.I.	Bootstrap (	Distance	Prob. DDH >= 70%
JMEV01000000	JOKB01000000	22	2.899647	22 - 22%	0.1992	0
JMEV01000000	ARTN00000000	20.2	2.888275	20.2 - 20.2	0.2178	0
JMEV01000000	ARND00000000	-9999			-9999	
JMEV01000000	JMEX01000000	22.9	2.935658	22.9 - 22.9	0.1912	0
JMEV01000000	JMEW01000000	22.4	2.904623	22.4 - 22.4	0.196	0
JMEV01000000	JMFF01000000	21.9	2.900571	21.9 - 21.9	0.2006	0
JMEV01000000	JMFE01000000	22.1	2.905587	22 - 22.1%	0.1989	0
JMEV01000000	BAOX01000000	46.5	3.124931	46.5 - 46.5	0.0803	10.83
JMEV01000000	NZ_CP010797.1	42.4	3.220153	42.4 - 42.4	0.0917	4.82
JMEV01000000	JMFC01000000	43.3	3.250196	43.3 - 43.3	0.089	5.85
JMEV01000000	JMFD01000000	32.9	3.084305	32.9 - 32.9	0.1274	0.32
JMEV01000000	JMFA01000000	33	3.070991	33 - 33%	0.1272	0.33
JMEV01000000	JMEZ01000000	33	3.086276	33 - 33%	0.127	0.33
JMEV01000000	JMEM01000000	34	3.102428	34 - 34%	0.1226	0.47
JMEV01000000	JZIS01000000	28.1	3.025618	28.1 - 28.1	0.1532	0.04
JMEV01000000	AXVF00000000	27.9	3.021006	27.9 - 27.9	0.1541	0.04
JMEV01000000	LFDS01000000	28	3.026574	28 - 28%	0.1536	0.04
JMEV01000000	JMEY01000000	29.3	3.039474	29.3 - 29.3	0.1462	0.08
JMEV01000000	JMEU01000000	29	3.043245	29 - 29%	0.1478	0.07
JMEV01000000	JMES01000000	27.9	3.019653	27.9 - 27.9	0.1542	0.04
JMEV01000000	JMER01000000	28.1	3.016526	28.1 - 28.1	0.1533	0.04
JMEV01000000	JMET01000000	29.2	3.062625	29.2 - 29.2	0.1467	0.07
JMEV01000000	JMEQ01000000	27.9	3.019977	27.9 - 27.9	0.1545	0.04

JMEV01000000	JMEP01000000	29.2	3.061572	29.2 - 29.2	0.1465	0.08
JMEV01000000	JMEO01000000	29.1	3.03946	29.1 - 29.1	0.1474	0.07
JMEV01000000	JMEN01000000	29	3.035506	29 - 29%	0.1477	0.07
JMEV01000000	CAPS00000000	30.7	3.018599	30.7 - 30.7	0.1381	0.14
Group E2-4						
Query genome	Reference genome	DDH	Model C.I.	Bootstrap (	Distance	Prob. DDH >= 70%
BAOX01000000	JOKB01000000	21.3	2.821507	21.3 - 21.3	0.2064	0
BAOX01000000	ARTN00000000	20.9	2.826377	20.9 - 21%	0.2097	0
BAOX01000000	ARND00000000	-9999			-9999	
BAOX01000000	JMEX01000000	21.2	2.825755	21.2 - 21.3	0.2066	0
BAOX01000000	JMEW01000000	21.3	2.817231	21.3 - 21.3	0.2058	0
BAOX01000000	JMFF01000000	21	2.815155	21 - 21%	0.2092	0
BAOX01000000	JMFE01000000	21.4	2.825742	21.4 - 21.4	0.2054	0
BAOX01000000	JMEV01000000	46.5	3.124931	46.5 - 46.5	0.0803	10.83
BAOX01000000	NZ_CP010797.1	45	3.200654	45 - 45%	0.0844	8.17
BAOX01000000	JMFC01000000	45	3.157344	45 - 45%	0.0842	8.25
BAOX01000000	JMFD01000000	31.8	3.033918	31.8 - 31.8	0.1327	0.22
BAOX01000000	JMFA01000000	32.1	3.027924	32.1 - 32.1	0.1315	0.24
BAOX01000000	JMEZ01000000	31.9	3.02665	31.9 - 31.9	0.132	0.23
BAOX01000000	JMEM01000000	31.9	3.02298	31.9 - 31.9	0.1321	0.23
BAOX01000000	JZIS01000000	28.7	3.010007	28.7 - 28.7	0.1496	0.06
BAOX01000000	AXVF00000000	28.5	3.006439	28.5 - 28.5	0.1505	0.06
BAOX01000000	LFDS01000000	28.4	3.008569	28.4 - 28.4	0.1511	0.05
BAOX01000000	JMEY01000000	28	3.006953	28 - 28%	0.1539	0.04
BAOX01000000	JMEU01000000	28	3.005871	28 - 28%	0.1538	0.04
BAOX01000000	JMES01000000	28.7	3.007533	28.7 - 28.7	0.1497	0.06
BAOX01000000	JMER01000000	28.6	3.00579	28.6 - 28.6	0.15	0.06
BAOX01000000	JMET01000000	28.1	3.005423	28.1 - 28.1	0.1529	0.05
BAOX01000000	JMEQ01000000	28.2	3.005574	28.2 - 28.2	0.1526	0.05
BAOX01000000	JMEP01000000	28.1	3.005423	28.1 - 28.1	0.153	0.05
BAOX01000000	JMEO01000000	28.1	3.005776	28.1 - 28.1	0.1529	0.05
BAOX01000000	JMEN01000000	28	3.00574	28 - 28%	0.1539	0.04
BAOX01000000	CAPS00000000	31.1	3.007535	31.1 - 31.1	0.1361	0.17
Group E2-5						
Query genome	Reference genome	DDH	Model C.I.	Bootstrap (	Distance	Prob. DDH >= 70%
NZ_CP010797.1	JOKB01000000	20.2	2.875993	20.2 - 20.2	0.2175	0
NZ_CP010797.1	ARTN00000000	20.1	2.903683	20.1 - 20.1	0.2183	0
NZ_CP010797.1	ARND00000000	-9999			-9999	
NZ_CP010797.1	JMEX01000000	20.2	2.892591	20.2 - 20.2	0.2179	0
NZ_CP010797.1	JMEW01000000	20.3	2.886779	20.3 - 20.3	0.2168	0
NZ_CP010797.1	JMFF01000000	20.2	2.886077	20.1 - 20.2	0.2181	0
NZ_CP010797.1	JMFE01000000	20.2	2.885661	20.2 - 20.2	0.2173	0
NZ_CP010797.1	JMEV01000000	42.4	3.220153	42.4 - 42.4	0.0917	4.82
NZ_CP010797.1	BAOX01000000	45	3.200654	45 - 45%	0.0844	8.17
NZ_CP010797.1	JMFC01000000	87.2	2.909668	87.2 - 87.2	0.0152	94.75
NZ_CP010797.1	JMFD01000000	31.6	3.177853	31.6 - 31.6	0.1339	0.2
NZ_CP010797.1	JMFA01000000	31.5	3.121973	31.4 - 31.5	0.1345	0.19
NZ_CP010797.1	JMEZ01000000	31.5	3.148921	31.5 - 31.5	0.1343	0.19

NZ_CP010797.1	JMEM01000000	31.4	3.133552	31.4 - 31.4	0.1346	0.19
NZ_CP010797.1	JZIS01000000	27.5	3.077759	27.5 - 27.5	0.157	0.03
NZ_CP010797.1	AXVF00000000	27.4	3.062715	27.4 - 27.4	0.1576	0.03
NZ_CP010797.1	LFDS01000000	27.4	3.083969	27.4 - 27.4	0.1576	0.03
NZ_CP010797.1	JMEY01000000	27.4	3.026672	27.4 - 27.4	0.1576	0.03
NZ_CP010797.1	JMEU01000000	27.2	3.027126	27.2 - 27.2	0.1591	0.03
NZ_CP010797.1	JMES01000000	27.3	3.041463	27.3 - 27.3	0.1579	0.03
NZ_CP010797.1	JMER01000000	27.3	3.032581	27.3 - 27.3	0.158	0.03
NZ_CP010797.1	JMET01000000	27.4	3.057615	27.4 - 27.4	0.1573	0.03
NZ_CP010797.1	JMEQ01000000	27.3	3.057156	27.3 - 27.3	0.1582	0.03
NZ_CP010797.1	JMEP01000000	27.4	3.056768	27.4 - 27.4	0.1575	0.03
NZ_CP010797.1	JMEO01000000	27.2	3.031559	27.2 - 27.2	0.1586	0.03
NZ_CP010797.1	JMEN01000000	27.3	3.030267	27.3 - 27.3	0.1584	0.03
NZ_CP010797.1	CAPS00000000	29.6	3.022032	29.6 - 29.7	0.1441	0.09
Group E2-6						
Query genome	Reference genome	DDH	Model C.I.	Bootstrap (	Distance	Prob. DDH >= 70%
JMFD01000000	JOKB01000000	20.6	2.840616	20.6 - 20.6	0.2134	0
JMFD01000000	ARTN00000000	20.4	2.854134	20.4 - 20.4	0.2157	0
JMFD01000000	ARND00000000	-9999			-9999	
JMFD01000000	JMEX01000000	20.5	2.848266	20.5 - 20.5	0.2142	0
JMFD01000000	JMEW01000000	20.6	2.849756	20.6 - 20.6	0.2134	0
JMFD01000000	JMFF01000000	20.4	2.845946	20.4 - 20.4	0.2149	0
JMFD01000000	JMFE01000000	20.6	2.854172	20.6 - 20.6	0.2138	0
JMFD01000000	JMEV01000000	32.9	3.084305	32.9 - 32.9	0.1274	0.32
JMFD01000000	BAOX01000000	31.8	3.033918	31.8 - 31.8	0.1327	0.22
JMFD01000000	NZ_CP010797.1	31.6	3.177853	31.6 - 31.6	0.1339	0.2
JMFD01000000	JMFC01000000	31.5	3.132992	31.5 - 31.5	0.1342	0.19
JMFD01000000	JMFD01000000	100	0.002646	100 - 100%	0	98.3
JMFD01000000	JMFA01000000	87	3.114755	87 - 87%	0.0154	94.64
JMFD01000000	JMEZ01000000	69.6	3.294275	69.6 - 69.6	0.0367	77.55
JMFD01000000	JMEM01000000	68	3.345255	68 - 68%	0.039	74.38
JMFD01000000	JZIS01000000	28.1	3.056643	28.1 - 28.1	0.1534	0.04
JMFD01000000	AXVF00000000	28.1	3.050903	28.1 - 28.1	0.1531	0.05
JMFD01000000	LFDS01000000	28.3	3.081406	28.3 - 28.3	0.1522	0.05
JMFD01000000	JMEY01000000	28.5	3.041157	28.5 - 28.5	0.1507	0.05
JMFD01000000	JMEU01000000	28.1	3.032277	28.1 - 28.1	0.1533	0.04
JMFD01000000	JMES01000000	28.2	3.045261	28.2 - 28.2	0.1525	0.05
JMFD01000000	JMER01000000	28.1	3.034748	28.1 - 28.1	0.1531	0.05
JMFD01000000	JMET01000000	28.2	3.045535	28.2 - 28.2	0.1527	0.05
JMFD01000000	JMEQ01000000	28	3.043152	28 - 28%	0.1535	0.04
JMFD01000000	JMEP01000000	28.2	3.044245	28.2 - 28.2	0.1527	0.05
JMFD01000000	JMEO01000000	28	3.029697	28 - 28%	0.1538	0.04
JMFD01000000	JMEN01000000	28	3.027513	28 - 28%	0.1535	0.04
JMFD01000000	CAPS00000000	30.9	3.022337	30.9 - 30.9	0.1375	0.15
Group E2-7						
Query genome	Reference genome	DDH	Model C.I.	Bootstrap (	Distance	Prob. DDH >= 70%
JMEN01000000	JOKB01000000	22.6	2.898779	22.6 - 22.6	0.1942	0
JMEN01000000	JMEY01000000	80.8	3.204293	80.8 - 80.8	0.0225	91.16

JMEN01000000	JMFD01000000	28	3.027513	28 - 28%	0.1535	0.04
JMEN01000000	JMEX01000000	23.1	2.906309	23.1 - 23.1	0.1896	0
JMEN01000000	JMEW01000000	22.5	2.893871	22.5 - 22.5	0.1946	0
JMEN01000000	JMEU01000000	80.5	2.969147	80.5 - 80.5	0.0227	90.98
JMEN01000000	JMES01000000	78.2	3.267375	78.2 - 78.2	0.0255	89.1
JMEN01000000	JMER01000000	79.3	3.320445	79.3 - 79.3	0.0242	90.03
JMEN01000000	JMFF01000000	22.2	2.889654	22.2 - 22.2	0.1977	0
JMEN01000000	JMFE01000000	22.6	2.898558	22.6 - 22.6	0.1937	0
JMEN01000000	JMFC01000000	28.2	3.04425	28.2 - 28.2	0.1523	0.05
JMEN01000000	JMFA01000000	28.8	3.051789	28.8 - 28.8	0.1488	0.06
JMEN01000000	JMEZ01000000	28.5	3.02761	28.5 - 28.5	0.1508	0.05
JMEN01000000	JMEV01000000	29	3.035506	29 - 29%	0.1477	0.07
JMEN01000000	JMET01000000	80.7	2.688965	80.7 - 80.7	0.0225	91.12
JMEN01000000	JMEQ01000000	81.4	3.111312	81.4 - 81.4	0.0218	91.58
JMEN01000000	JMEP01000000	80.7	2.692911	80.7 - 80.7	0.0225	91.13
JMEN01000000	JMEO01000000	82.4	2.724175	82.4 - 82.4	0.0206	92.26
JMEN01000000	JMEM01000000	29.4	3.048811	29.4 - 29.4	0.1453	0.08
JMEN01000000	LFDS01000000	79.1	3.080221	79.1 - 79.1	0.0244	89.9
JMEN01000000	ARTN00000000	20.3	2.869777	20.3 - 20.4	0.216	0
JMEN01000000	ARND00000000	-9999			-9999	
JMEN01000000	AXVF00000000	80.8	2.862236	80.8 - 80.8	0.0224	91.17
JMEN01000000	CAPS00000000	26.9	3.01071	26.9 - 26.9	0.1606	0.03
JMEN01000000	BAOX01000000	28	3.00574	28 - 28%	0.1539	0.04
JMEN01000000	JZIS01000000	80.1	3.123756	80.1 - 80.1	0.0233	90.65
JMEN01000000	NZ_CP010797.1	27.3	3.030267	27.3 - 27.3	0.1584	0.03
Group E2-8						
Query genome	Reference genome	DDH	Model C.I.	Bootstrap (	Distance	Prob. DDH >= 70%
CAPS00000000	JOKB01000000	21.6	2.830852	21.6 - 21.6	0.2032	0
CAPS00000000	ARTN00000000	20.6	2.828702	20.6 - 20.6	0.2136	0
CAPS00000000	ARND00000000	-9999			-9999	
CAPS00000000	JMEX01000000	21.5	2.839691	21.5 - 21.5	0.2038	0
CAPS00000000	JMEW01000000	21.5	2.840446	21.5 - 21.5	0.2042	0
CAPS00000000	JMFF01000000	21.1	2.835341	21.1 - 21.1	0.2083	0
CAPS00000000	JMFE01000000	21.6	2.84452	21.6 - 21.6	0.2032	0
CAPS00000000	JMEV01000000	30.7	3.018599	30.7 - 30.7	0.1381	0.14
CAPS00000000	BAOX01000000	31.1	3.007535	31.1 - 31.1	0.1361	0.17
CAPS00000000	NZ_CP010797.1	29.6	3.022032	29.6 - 29.7	0.1441	0.09
CAPS00000000	JMFC01000000	29.7	3.015213	29.7 - 29.7	0.1437	0.09
CAPS00000000	JMFD01000000	30.9	3.022337	30.9 - 30.9	0.1375	0.15
CAPS00000000	JMFA01000000	30.9	3.024549	30.9 - 30.9	0.1374	0.15
CAPS00000000	JMEZ01000000	30.8	3.015266	30.8 - 30.8	0.1379	0.15
CAPS00000000	JMEM01000000	30.8	3.01487	30.8 - 30.8	0.1377	0.15
CAPS00000000	JZIS01000000	27.1	3.015674	27.1 - 27.1	0.1597	0.03
CAPS00000000	AXVF00000000	26.9	3.013666	26.9 - 26.9	0.1605	0.03
CAPS00000000	LFDS01000000	26.9	3.01572	26.9 - 26.9	0.1609	0.02
CAPS00000000	JMEY01000000	26.8	3.005878	26.8 - 26.8	0.1615	0.02
CAPS00000000	JMEU01000000	26.8	3.007005	26.8 - 26.8	0.1616	0.02
CAPS00000000	JMES01000000	26.9	3.011975	26.9 - 26.9	0.1606	0.03
CAPS00000000	JMER01000000	27.1	3.012532	27.1 - 27.1	0.1593	0.03

CAPS00000000	JMET01000000	26.9	3.013386	26.9 - 26.9	0.161	0.02
CAPS00000000	JMEQ01000000	26.6	3.009438	26.6 - 26.6	0.1626	0.02
CAPS00000000	JMEP01000000	26.9	3.012704	26.9 - 26.9	0.1609	0.02
CAPS00000000	JMEO01000000	26.9	3.009852	26.9 - 26.9	0.1606	0.03
CAPS00000000	JMEN01000000	26.9	3.01071	26.9 - 26.9	0.1606	0.03
Group F-1						
Query genome	Reference genome	DDH	Model C.I.	Bootstrap (	Distance	Prob. DDH >= 70%
LPZL00000000	JMLQ01000000	21.3	2.34	21.3 - 21.3	0.2064	0
LPZL00000000	JXQS01000000	20.6	2.32	20.6 - 20.6	0.2134	0
LPZL00000000	LPZO00000000	43.7	2.54	43.7 - 43.7	0.088	6.32
Group G-3						
Query genome	Reference genome	DDH	Model C.I.	Bootstrap (	Distance	Prob. DDH >= 70%
AODO00000000	JMLO01000000	21.2	2.921555	21.2 - 21.2	0.2073	0
AODO00000000	CVQP01000000	27.7	3.020569	27.7 - 27.7	0.1556	0.04

Supplementary Table 4. A list of group A specific genes

**Locus tag**	**Function**	**Protein Sequence**	**Nucleotide sequence**
ch56_peg917	hypothetical protein	MRIRHLIAAVPVALLVLTGCSDG<br>GDSDPKDTKNDAGETTITAEAG<br>ADAQETAVSVMCAQVVAYLDA<br>TQQQAESTGQPFDRDAKGAEFL<br>DMLKANGGTVVAAYNQQAVA<br>AGEPTLDPATATWEDVPAEARE<br>LIEQSVQSGISGSC	atgcgcattcgacatctgatcgccgccgtccccgtcgccctgctggtcctcaccggctgcagtgacg<br>gtggcgactcggacccgaaggacaccaagaacgacgcgggcgagaccaccatcacggccgagg<br>ccggcgccgacgcccaggagaccgcggtctcggtgatgtgcgcgcaggtggtggcctacctcgac<br>gcgacgcagcagcaggccgagagcaccgggcagccgttcgaccgggatgcgaagggcgccgag<br>ttcctcgacatgctgaaggcgaacggcggcaccgtggtggcggcgtacaaccagcaggcggtgg<br>cggccggtgagccgacgctcgatccggcgaccgcgacgtgggaggacgtgccggccgaggcccg<br>tgagctcatcgagcagtcggtgcagtccgggatctccggcagctgctga
ch56_peg2696	hypothetical protein	MIDDADITAHLTDEVRALPYPGR<br>WSNPPFAGDGAAVIARDGDLTE<br>LRVDRRAGRVDWVDLQTGAGQ<br>MLAPSLRVLGALAAAYATALRDT<br>RGAGDRALRRIERTFLDSVRTVS<br>ADLAADGTFWATAAEELGDGVL<br>GGDDLPEPLTVVPAGGSTVVIA<br>MPMLRLHRALADEGLTLSDYRS<br>AVSYVTLSDDLDTTLANTARPGA<br>QASGPARVLVVDGQSPLTADAL<br>AFPALRTLILVEPATLPEHLAAAA<br>AALAVVTVGPREPFARIADLLAH<br>HAV	atgatcgacgacgccgacatcacggcgcacctgaccgacgaggtgcgggcgctcccctatccggg<br>gcggtggtcgaatcctccgttcgcgggtgacggcgcggcggtgatcgcccgcgacggcgacctga<br>cggagttgcgcgtcgaccggcgggccggtcgcgtcgactgggtggatctgcagaccggagccggg<br>cagatgctcgcaccgtcgctgcgcgtcctcggggcgctcgcggcggcgtacgcgacagcattacg<br>ggacacccgaggagccggcgaccgcgcgctgcgtcgcatcgagcggacgttcctcgattccgtcc<br>gcacggtgtccgccgatctggccgccgacggcacgttctgggccaccgcggcggaggaactcggc<br>gacggtgtgctcggcggtgacgacctccccgaaccgctcaccgtggtaccggcgggcgggtcgac<br>cgtcgtcatcgcgatgccgatgctgcggctgcaccgggcgctcgccgacgagggactcaccctgtc<br>cgactaccgctccgcggtgtcgtacgtgacgctgtccgacgacctcgacaccacactggcgaaca<br>cagcccgccccggtgcacaggcgtcgggaccggcccgggtgctcgtcgtcgacgggcagagccc<br>gctcaccgccgacgccctcgcctttccggcgctacggacgctgatcctcgtcgagcccgccacact<br>gccggagcatctcgccgcagccgccgcggccctcgcggtggtgaccgtcggcccccgcgaaccgt<br>tcgcccggatcgccgatctcctcgcccaccacgcagtctga

Locus tag	Function	Protein Sequence	Nucleotide sequence
ch56_peg2789	hypothetical protein	MAGGRKRHVAGAAAAGALAVAGTFGAAPAAATVTGQGDVRTFGNSILVTMLNIDSTTDAPTRCATVVFDTAGRPVITDLQEIYDGGLGIWWTTMPTETLHLAGQYTGSADYRVTTECRDDDGVVRLADTVVTLPEGELVGNPPRYSELFGPF	atggccggaggacggaaacggcacgtcgcgggggccgccgcggcgggtgcactcgcggtggccgggacattcggtgcagcgccggccgcggcgacggtgaccgggcagggcgacgtgcgcaccttcggcaactcgatcctcgtgacgatgctgaacatcgactccaccaccgacgcaccgacgcggtgcgcgaccgtcgtcttcgacaccgccggccgacccgtgatcaccgatctgcaggagatctacgacggtggactcggcatctggtggaccacgatgcccaccgagacactccatctcgcggggcagtacacgggcagcgccgattaccgcgtcaccaccgagtgccgggacgacgacggtgtcgtgcgactggccgacaccgtcgtcaccctgcccgagggcgaactcgtcgggaacccgccccggtactcggaactgttcgggccgttctag
ch56_peg2885	hypothetical protein	LDRRFHDRFLHVFGRRLIGHVFSSSPRARPSVCLSNVTRPRGGDGCVLVPRARDCLVSLHVLHSVRYHCRIARCLAGWRRSTAHAPDRSRPERLLIQRASRDRDYLY	ttggaccgacgcttccacgatcggttcctgcacgttttcgggcggcgcttgatcggccacgtattctcctcgagcccccgggcgcgtccatccgtctgtctgtcgaatgtgacgcggccgcgcgggggcgatggttgtgtactcgtacctcgggcacgagattgtctggtctcgctccacgtactccacagtgtccgttatcactgcagaatcgcgcggtgcctggctggatggcggcgatccacggcgcatgcgcctgaccgttcacggcccgagcgcctgctcatccagcgtgcctcacgtgacagggattacctgtactga
ch56_peg4192	hypothetical protein	MNRQTIEHRGWVVAAAAATAVASGLLGAAVAGGDDQAESNPATLTTEEEGTDSPEDPSGLEQTNSFGYIPEHETTLETDPIGPEGGGIEDDPFELGGGRIDPAVVVPVPADATLTTPAG	atgaatcgacagacgatcgagcaccgcggctgggtggtcgcggcagcggccgccacggccgtcgcatcgggactgctcggggccgccgtcgccggaggcgacgaccaggccgagtcgaatcccgccacgctcaccaccgaggaggaaggcaccgactcgccggaggacccgagcggtctcgagcagaccaattccttcggctacatccccgagcacgagacgaccctcgagaccgatcccatcggaccggagggtggcggcatcgaggacgacccgttcgaactcggcggcgggcgcatcgacccggccgtcgtcgtcccggtgcccgcagacgcgaccctcaccaccccggccggttga
